# Gel-Dominated Microstructural Evolution and Strength Development in Phosphogypsum-Based PRBA Systems for Road Base Applications

**DOI:** 10.3390/gels12080726

**Published:** 2026-08-15

**Authors:** Ruiyuan Li, Guangdong An, Yuwen Deng, Yonglan Zong, Kai Li, Xiaofeng Huang, Ping Ning, Xin Sun, Quxiu Dai

**Affiliations:** 1 Faculty of Environmental Science and Engineering, Kunming University of Science and Technology, Kunming 650500, China; lry18687227111@163.com (R.L.); 18687136575@163.com (G.A.); yuwen_ddd@163.com (Y.D.); 15887980249@163.com (Y.Z.); likaichi@163.com (K.L.); hxfhgxy@kust.edu.cn (X.H.); ningping58@sina.com (P.N.); 2 Department of Chemistry, University of Toronto, Toronto, ON M5S 3H6, Canada

**Keywords:** phosphogypsum, fly ash, steel slag, powdered recycled binder–aggregate, composite activation, hydration kinetic model

## Abstract

A significant pile-up of phosphogypsum, with utilization under 30% in some areas, has caused serious environmental problems. This study presents a powdered recycled binder-aggregate (PRBA), prepared by activating a phosphogypsum-fly ash-steel slag ternary system via a sodium silicate-calcium hydroxide activator. The optimal L3 at 55% PRBA with PG:FA:SS of 4:3:4 achieved 28-day compressive strength of 9.33 MPa, 22.0% higher than control L0 at 7.65 MPa, reaching 12.47 MPa at 56 days. The C-S-H gel network evolved from 100–200 nm tubular to under 10 nm lamellar structures, reducing average pore diameter from 82 nm to 52.5 nm. Non-isothermal kinetic modeling revealed that C-S-H/AFt dehydration follows Jander three-dimensional diffusion with R^2^ = 0.998 and activation energy decreasing from 161.3 to 99.3 kJ/mol at 14 days. A relay-race mechanism was identified: steel slag provides early Ca^2+^, phosphogypsum supplies SO_4_^2−^ forming AFt skeletons, and fly ash densifies the matrix via pozzolanic reactions. Environmental assessment showed P and F solidification rates of 66.7% and 88.3%, plus 10.77 g CO_2_/kg carbonation. Converting inert industrial wastes into a reactive cementitious system enables 55% natural aggregate replacement and pollutant immobilization, offering scalable pathways for phosphogypsum valorization in road base applications.

## 1. Introduction

The annual production of bulk industrial solid waste worldwide has exceeded 10 billion tons, with cumulative stockpiles surpassing 100 billion tons. This massive accumulation occupies substantial land resources and creates severe environmental pressures across both developed and developing economies [1]. Phosphogypsum is a major byproduct of wet-process phosphoric acid production. Annual global emissions exceed 300 million tons. In most phosphate-producing regions, the comprehensive utilization rate remains below 30%. Long-term stockpiling not only leads to land occupation but also presents ecological threats. Recent comprehensive reviews have highlighted that phosphogypsum-based building materials offer promising high-value utilization pathways, yet their development remains constrained by hydration kinetic mismatches and long-term environmental risk uncertainties [2]. The soluble phosphorus and fluorine contaminants in PG can migrate with rainwater infiltration, posing persistent risks to surrounding soils and groundwater [3,4]. Natural sand and gravel aggregate resources are becoming depleted globally. Excessive mining has triggered severe ecological damage. This damage affects multiple continents. In Asia, river ecosystems are degrading. In Africa, biodiversity is being lost. In South America, landscapes are being destroyed. Utilizing industrial solid waste to prepare PRBAs as substitutes for natural sand and stone has become a critical pathway for achieving “dual carbon” goals and green sustainable development in civil engineering worldwide. The mutual remediation of CO_2_ emissions and industrial wastewater represents an emerging strategy for sustainable industrial development, offering synergistic benefits between carbon sequestration and wastewater treatment [5].

Existing research on recycled aggregates has primarily focused on construction waste, with significant contributions from research groups in Europe, North America, and East Asia [6,7,8]. These aggregates are relatively mature in technology and have achieved large-scale engineering applications. However, they are essentially inert or low-activity materials that mainly serve physical filling functions [9,10]. Research has shown that old mortar attached to recycled aggregate surfaces and microcracks result in high crushing indices and water absorption rates. These characteristics create weak interfacial transition zones (ITZs) with cement paste, causing PRBA concrete strength to decrease by 10–30% compared to natural sand concrete, with significantly deteriorated durability. Therefore, breaking through the limitations of conventional recycled aggregates that emphasize “replacement over activation” and developing new PRBAs with both filling and cementitious functions has become an important research direction [7,8,11]. Emerging studies on full-component solid waste cementitious materials further emphasize that carbon emission reduction and resource synergistic effects can be simultaneously achieved through rational ternary or multi-source solid waste mix proportion optimization [12].

In recent years, powdered recycled binder-aggregate with gelation and self-binding properties (PRBA) prepared from industrial solid wastes have gradually attracted academic attention globally. In this study, “powdered recycled binder-aggregate” (PRBA) refers to finely ground industrial solid waste (particle size < 0.28 mm) with latent cementitious reactivity. PRBAs serve dual functions as micro-aggregates and supplementary cementitious materials (SCMs). This distinguishes them from conventional construction and demolition (C&D) recycled aggregates. C&D aggregates are inert particulate materials. They serve only physical replacement functions. The PRBAs used here-phosphogypsum (PG), fly ash (FA), and steel slag (SS)-were sieved through a 50-mesh (0.297 mm) standard sieve. We emphasize that this ternary system constitutes a calcium-rich alkali-activated composite rather than a geopolymer. In conventional geopolymers, the primary binding phase is an aluminosilicate gel (N-A-S-H) formed under high-alkali, low-calcium conditions. In contrast, the present system generates calcium-silicate-hydrate (C-S-H) gel as the primary binding phase, accompanied by AFt crystalline products, which is characteristic of calcium-rich alkali-activated materials. It should be noted that the 28-day compressive strength of L3 (9.33 MPa) classifies this material as a road base or stabilized fill material. It is not structural concrete. Steel slag, rich in C_2_S, C_3_S, and free CaO, releases Ca^2+^ in alkaline environments and generates a calcium-silicate-hydrate (C-S-H) gel network, thereby providing early strength support. Under hydroxide ion attack, the fly ash glass phase continuously releases active silicon and aluminum. This triggers later-stage pozzolanic reactions that densify the structure. Phosphogypsum, rich in SO_4_^2−^, reacts with active aluminum to generate AFt (AFt, calcium aluminate trisulfate hydrate, Ca_6_[Al(OH)_6_]_2_(SO_4_)_3_·26H_2_O), an important hydration gel product that enhances strength through gel binding and fixation, forming early strength skeletons and provides sulfate activation effects. However, current research is mostly limited to single solid waste systems or simple binary combinations. There is a lack of systematic understanding regarding temporal reaction matching and activity synergy mechanisms of multi-source solid wastes in complex systems. Particularly, the problem of competitive reaction consumption caused by dissolution kinetic differences among different components has not been resolved.

Specifically, the large-scale application of phosphogypsum in the PRBA field still faces three major bottlenecks:

First, environmental risks are prominent. Globally, the leaching concentrations of phosphorus and fluorine in phosphogypsum typically range from 0.2 to 0.5 mg/L and 0.1 to 0.3 mg/L, respectively. These values depend on phosphate rock origin and acidulation processes. Traditional physical stockpiling cannot achieve chemical stabilization of pollutants. Simple mixing methods also cannot achieve this. Leakage from storage sites poses continuous threats. These threats affect soil and water bodies. Meskini et al. [13] demonstrated that lime-fly ash-treated phosphogypsum road materials can achieve heavy metal fixation rates of 25–100% and reduce radioactivity by 82%. Peng et al. [14] further achieved deep solidification of soluble phosphorus and fluorine in phosphogypsum through the Ca(OH)_2_-Al(OH)_3_ system, reducing PO_4_^3−^ concentration to 0.056 mg/L and elucidating the Ca^2+^-dominated precipitation solidification mechanism. Zhou et al. [15] investigated the stabilization/solidification mechanisms of tin tailings and fuming slag-based geopolymers for different heavy metals, revealing that cations are embedded in the aluminosilicate lattice while anions form insoluble precipitates, providing complementary insights for heavy metal immobilization in complex solid waste systems.

Second, resource utilization pathways are low end. Current applications mostly treat phosphogypsum as inert filling material. They utilize only its physical substitution effect. This is used to prepare gypsum-based cementitious materials [16]. It is also used for cement retarders. Concerning phosphogypsum, rich in SO_4_^2−^, the sulfate activation potential has not been fully developed [17]. This results in low product added value. It also causes small consumption capacity. Aggregate replacement rates are generally low. Research by the Kunming University of Science and Technology team [18] showed that paste filling technology using phosphogypsum as aggregate and multi-source solid wastes as cementitious materials achieved 19.5 MPa compressive strength at 60 days with 60% phosphogypsum content, with significantly reduced P, F, and heavy metal leaching. Li et al. [19] further confirmed that sustainable utilization of phosphogypsum in multi-solid waste recycled aggregate systems has significant environmental and economic benefits, still meeting C25 concrete mechanical performance requirements at 70% phosphogypsum content.

Third, synergistic mechanisms remain unclear. In the phosphogypsum-fly ash-steel slag ternary system, kinetic differences are significant. Steel slag shows fast dissolution. Phosphogypsum shows slow release. Fly ash shows slow reaction. Simple mixing can easily lead to excessive calcium ion generation. It can also cause formation of low-activity calcium silicate precipitates. These hinder hydration processes. The temporal mismatch of different hydration products can easily cause structural defects. Wu et al. [11] systematically studied the influence of phosphogypsum on hydration microstructure of alkali-activated slag, revealing the synergistic formation mechanism of the gel network and AFt [15]. Wang et al. [20] achieved 7-day strength increase from 0 to 20.2 MPa through nano-Aft-activated phosphogypsum–slag system, confirming the supporting role of AFt skeleton networks for early strength. International research on calcined phosphogypsum-based composite materials also proposed a “dual skeleton” synergistic mechanism of early dihydrate gypsum skeleton and later AFt + C-S-H skeleton. Shen et al. [21] demonstrated that steel slag-fly ash-phosphogypsum-solidified materials have excellent mechanical properties and environmental safety as subgrade materials. Weiksnar and Townsend [22] significantly improved chemical stability and leaching behavior by scientifically blending phosphogypsum with common aggregates.

Therefore, how to achieve “staggered complementarity” of multi-solid waste activities through composite activation and construct a technical pathway from “inert filling” to “active synergy” has become a key scientific issue for breaking through high-value utilization of phosphogypsum. Additionally, optimization of interfacial transition zones and improvement of micromechanical properties are also important directions that require attention in engineering applications. Research on synergistic hydration mechanisms of multi-solid waste systems will provide theoretical support for preparing high-performance PRBA subgrade concrete [23]. This study explores the engineering applicability of the developed material, with potential applications in prefabricated components, road infrastructure, and protective structures, as schematically illustrated in Figure S1, Supplementary Information, which depicts the complete technological chain from raw material preparation through composite activation and specimen fabrication to road base and prefabricated non-structural applications.

## 2. Results and Discussion

### 2.1. Mechanical Property Evolution of Aggregates

#### 2.1.1. Development Trend of Compressive Strength with Curing Age

**Figure 1:**
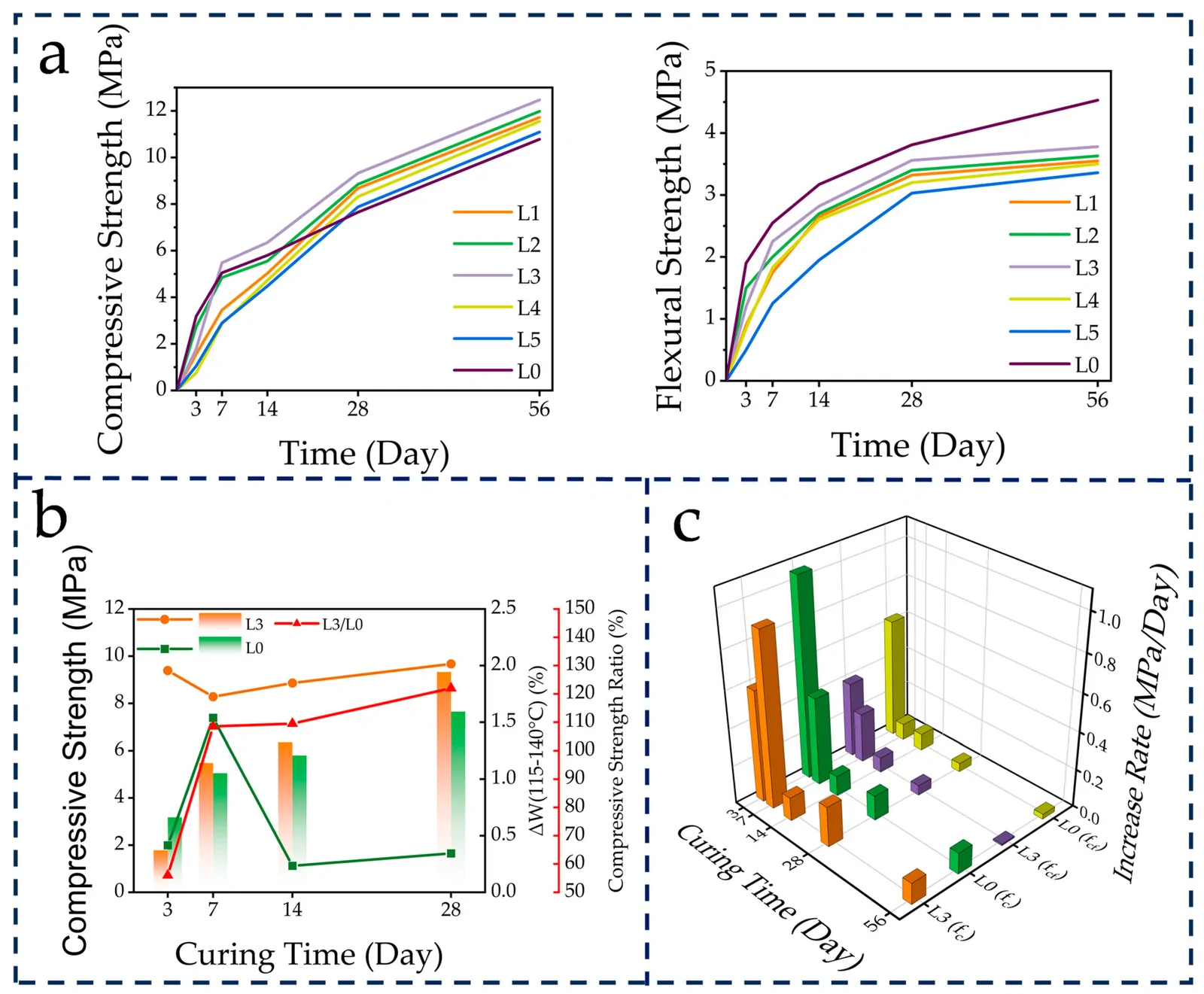
(**a**) Compressive and flexural strength curves of specimens cured for different ages; (**b**) compressive strength and the ratio of compressive strength of L0 and L3 cured at different ages, and the dehydration loss rate of C-S-H in the 115–140 °C temperature range; (**c**) average increase rates of compressive (f_c_) and flexural (f_cf_) strength under different curing ages for L0 and L3.

Figure 1a,b systematically reveal the strength evolution patterns of the six formulations from L0 to L5. The control group L0 exhibits typical cement-based material hydration characteristics [24,25,26,27,28], with 28-day compressive strength reaching 7.65 MPa and continuous growth to 10.78 MPa at 56 days. In terms of compressive strength, L0 demonstrates absolute advantages. This is in the very early stage of hydration. The hydration rate is fast. In flexural strength, L0 outperforms groups L1 to L5. This is throughout the entire curing period. The reason is fewer ITZ bonding deficiencies.

Group L1 exhibits fewer ITZ interface deficiencies and lower replacement rates; nevertheless, these advantages cannot compensate for the strength reduction arising from ITZ deficiencies. This is caused by ITZ deficiencies. The reason is insufficient active excitation strength phases in PRBAs. Therefore, 3-day strength is only 1.56 MPa. However, later fly ash pozzolanic effect compensation allows for recovery. At 28 days, strength recovers to 8.68 MPa. Group L2 has insufficient fly ash. This shows weakened later hydration processes. At 28 days, compressive strength is only 8.85 MPa.

Group L3 demonstrates obvious advantages. These are in compressive strength and hydration process optimization. This is achieved through its replacement rate and mix proportion. However, ITZ bonding deficiencies still cannot be compensated. Therefore, flexural strength defects persist. Group L4 has excessive fly ash. It lacks sufficient early strength advantages. This affects later strength. At 28 days, compressive strength is 8.32 MPa. Group L5 shows a 3-day compressive strength of 1.05 MPa. At 28 days, compressive strength recovers to 7.88 MPa. This is due to dense filling effects. However, 3-day flexural strength is only 0.5 MPa. This exposes fatal shortcomings. Interface defects occur under excessive replacement rates. Meanwhile, at a 60% replacement rate, obvious compressive strength decreases appear. These are cliff-like decreases. Therefore, designing replacement rates at 60% and below is reasonable.

Combined analysis of Figure 1b,c shows the results. At 7 days of curing, group L0 exhibits an abnormal surge. This is in the C-S-H weight loss temperature zone of 115 to 140 °C. Its higher weight loss rate indicates something. Hydration processes at this time have tended to completely enter the deceleration period. Meanwhile, L0 compressive strength development presents typical cement hydration laws. It reaches peak hydration rate at 3 days. Growth rates between ages are as follows. From 0 to 3 days, it is 1.06 MPa per day. From 3 to 7 days, it is 0.47 MPa per day. From 7 to 14 days, it is 0.11 MPa per day. From 14 to 28 days, it is 0.13 MPa per day. From 28 to 56 days, it is 0.11 MPa per day. This indicates that hydration tends to stagnate after 14 days. It maintains growth rates around 0.11 MPa per day.

Figure 1c shows the results. Group L3 presents a delayed hydration process. It also shows extended activity period characteristics. Growth rates between ages are as follows. They are 0.59 MPa per day, 0.93 MPa per day, 0.12 MPa per day, 0.20 MPa per day, and 0.11 MPa per day. This indicates that PRBA addition effectively controls early cement hydration rate. It also controls hydration process strength. An increase in compressive strength appears. This occurs during 14 to 28 days of hydration. This indicates that fly ash plays a key role. This role is in later hydration processes. It achieves the intended effect of fly ash addition. These delayed hydration characteristics and later-stage strength gains are consistent with recent findings in alkali-activated steel slag-GGBS-fly ash ternary systems, where optimized synergistic ratios achieved high strength and toughness through multi-level C-S-H gel network formation [29].

As shown in Figure 1b, L3’s 28-day strength reaches 9.33 MPa (122.0% of L0). Its 56-day strength reaches 12.47 MPa (115.7% of L0). These values demonstrate sustained hydration activity. However, L0 and L3 have different effective binder contents. L0 uses cement only. L3 uses cement plus reactive PRBAs. Thus, direct quantitative comparison is not equivalent. The value lies in demonstrating viability. PRBA concrete achieves competitive performance against conventional cement-sand concrete. This supports road base material applications. It does not claim superiority under equivalent binder conditions.

#### 2.1.2. Flexural Strength Development and Interface Influence Analysis

Figure 1a shows the results. In terms of flexural strength, group L0 reaches 3.81 MPa at 28 days. Group L3 reaches 3.56 MPa at 28 days. L3 flexural strength is the highest among experimental groups. However, it is still only 93.4% of L0. This indicates that PRBA-related defects are extremely significant. These are ITZ deficiency defects. They cannot be compensated. This is even through optimized replacement rates and mixed proportions. Therefore, differences in flexural strength among other experimental groups reflect strength contributions from different proportions of the three PRBAs. This “high compressive strength, low flexural strength” divergence phenomenon originates from the interface weakening effect introduced by PRBAs [8,9,27,28]. Fine powder aggregates improve compressive strength. This is through pore filling. However, they have high brittle characteristics. They also have extremely poor shear resistance. These decrease material fracture toughness. SEM interface morphology observation shows the results. Micro-gaps of 50 to 80 nm exist in the ITZ zone. These are in L3–28 specimens. However, AFt needle-like crystals show perpendicular interpenetration growth. This occurs at the interface. It compensates for partial ITZ bonding losses. However, this is still insufficient. It cannot compensate for the negative effects of ITZ deficiency.

Calculated through Equation (1), Supplementary Table S3 presents the brittleness index values, demonstrating that *B* calculations show that the L0 *B* value difference throughout the curing stage is 0.71, while L3 reaches 1.83. After L0 hydration peak, *B* values of groups L1–L5 are all greater than L0 group, confirming significant brittleness increase in PRBA concrete [30,31,32].

(1) $$B=\frac{f_{c}}{f_{cf}}$$

### 2.2. Reaction Product Formation and Gel Evolution

#### 2.2.1. Hydration Product Morphology and Evolution

**Figure 2:**
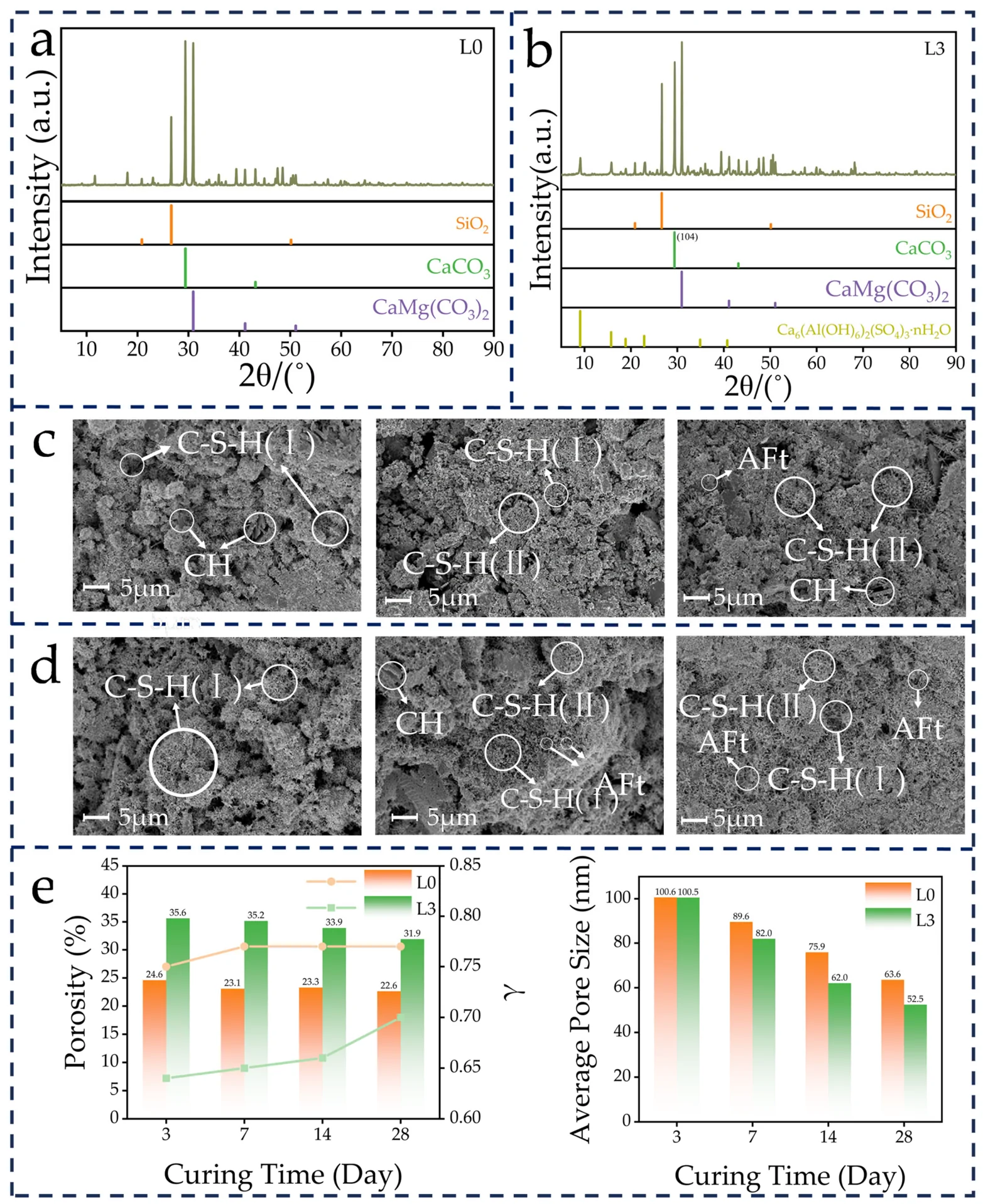
(**a**) XRD analysis of L0 cured for 28 days; (**b**) XRD analysis of L3 cured for 28 days; (**c**) SEM images of L0 after 3-day, 7-day, and 28-day curing; (**d**) SEM images of L3 after 3-day, 7-day, and 28-day curing; (**e**) apparent porosity and compaction index (γ) charts and average pore size charts for L0 and L3.

Figure 2a,b show the results. For the cement-based control L0, conventional hydration dominates; for the PRBA-containing groups, alkali-sulfate activation generates additional binding phases. When hydration proceeds to 28 days, no obvious Ca_6_(Al(OH)_6_)_2_(SO_4_)_3_·26H_2_O peak is detected in L0. However, obvious AFt peaks are detected in group L3. This indicates that PRBAs generate more alkali-activated products during the reaction process. In XRD analysis, both L0 and L3 28-day samples exhibited the characteristic CaCO_3_ (104) reflection at 29.4°; the (202) peak at 43.1° was also detectable in the full-range patterns (Figure 2a,b), albeit with significantly attenuated intensity relative to the (104) peak. The marked broadening of the (104) peak (FWHM = 0.098°) indicates nanocrystalline CaCO_3_ formation, as quantified by the Scherrer equation (Supplementary Table S4). This phenomenon results from calcium carbonate structure nanocrystallization. According to Scherrer effect theory, half-width and crystallite size satisfy a relationship.

(2) $$\beta =\frac{K\lambda }{D\cos\theta }$$

Scherrer constant K = 0.89. The Cu K-alpha radiation wavelength is 0.15406 nm. Theta is the Bragg angle. Peak fitting was performed. This was on L3 group 28-day sample XRD patterns. Calcium carbonate (104) crystal plane was measured. Two theta is 29.447 degrees. FWHM is 0.098 degrees. Integral Breadth is 0.146 degrees. Instrument broadening of 0.08 degrees was deducted. Substituting into the Scherrer formula yields crystallite sizes. Based on FWHM, this is approximately 140 nm. Based on Integral Breadth, this is approximately 66 nm. Details are in Supplementary Table S4. Integral Breadth is less sensitive to peak shape. It is more consistent with Scherrer formula definition. Therefore, approximately 66 nm is taken as the effective value.

This result indicates that carbonation products are submicron fine-crystal calcium carbonate with crystallite diameters of approximately 66 nm (Supplementary Table S4). While these are not ultra-fine nanocrystals (<20 nm), they are still significantly smaller than conventional carbonation products (>200 nm) and possess high specific surface area and reactivity. The pronounced peak broadening of the CaCO_3_ (104) reflection (FWHM = 0.098°) is consistent with the Scherrer effect, where crystallite refinement reduces the coherent diffraction domain size. In the full-range XRD patterns (Figure 2a,b), the (202) peak at 43.1° is detectable but exhibits markedly attenuated intensity relative to the (104) peak, further corroborating the nanocrystalline nature of the carbonation products. These submicron fine-crystals provide structural evidence for the material’s enhanced carbon sequestration capability (10.77 g CO_2_/kg) and improved matrix densification through nano-filling effects.

SEM images in Figure 2c,d present differences. These are in hydration product development. They compare L0 and L3 groups at different curing ages. After 3 days of curing, L0 group fracture surfaces were examined. They are only covered with sparse tubular C-S-H gel. Incompletely reacted flaky calcium hydroxide crystals are visible. This indicates that cement hydration is still in transition. It is moving from the induction period to the acceleration period. In contrast, the L3 group at the same age shows different features. It already shows high-density nanofiber-like C-S-H interwoven networks. Tubular structure diameters are concentrated in the 100 to 200 nm range. This rapid nucleation phenomenon originates from the reaction between sodium silicate-derived silicate ions and calcium ions released from steel slag dissolution. Silicate ions are produced by sodium silicate hydrolysis. Calcium ions are released from steel slag dissolution. These rapidly react in strong alkaline environments. This shortens the induction time. This time is required for fly ash glass phase structure depolymerization. Notably, calcium hydroxide supersaturated precipitation in the L3 group is significantly less than in the L0 group. This indicates that composite activators effectively promote calcium ion consumption and transformation. This avoids later-stage alkali-silica reaction risks.

**Figure 3:**
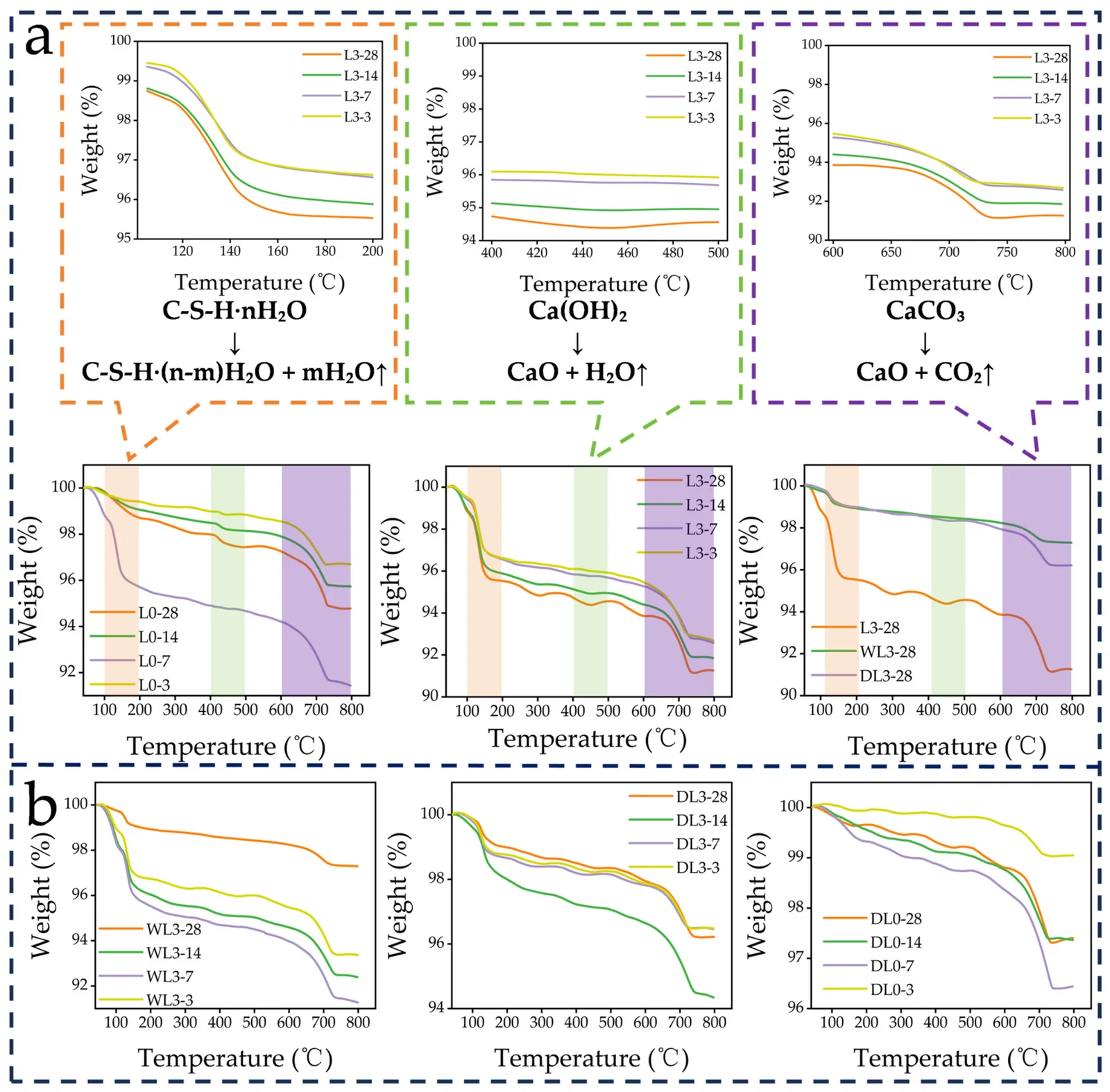
(**a**) TG-DSC analysis diagrams of L0, L3, and L3 under washing and selective dissolution treatment, along with enlarged views of the key weight loss temperature regions (115–140 °C, C-S-H/AFt; 405–450 °C, CH; 650–750 °C, CaCO_3_); (**b**) TG curves of L3 and L0 materials of different ages after separation of hydrated products were separated using deionized water and selective dissolution.

Figure 2c shows the results. When cured to 7 days, tubular structures on the L0 group surfaces basically disappear. They transform into coral-like Type II C-S-H gel. This marks the transition of hydration reaction. It moves from surface nucleation control to internal diffusion control. However, the L3 group still retains large amounts of tubular C-SH at this time. Multi-level sheet-like cross-linking structures appear at their roots. These have thickness of less than 10 nm. Obvious tube-tube bridging characteristics appear at their heads. This unique “tube-sheet-bridge” multi-level structure originates from SO_4_^2–^ continuously dissolved from phosphogypsum reacting with active Al^3+^ from fly ash to generate AFt crystals. Their needle-like morphology interpenetrates into C-S-H gel skeletons to form interlocking effects. This structure not only provides support for early strength but also absorbs large amounts of free water through capillary action. The abnormal weight loss peak before 100 °C in L3–7 water-washed samples in Figure 3a confirms this structure’s extremely strong water confinement capability [32].

As shown in Figure 2d, morphology evolution at 28 days further reflects L3 group’s long-term hydration advantages. Although the L0 group surfaces are covered with dense C-S-H, newly generated products only attach to original gel layer surfaces, with slow thickness growth, reflecting reactant supply tending to depletion. In contrast, the L3 group presents large-scale sheet-like C-S-H dense stacking. Gel phases completely encapsulate unreacted particles. This forms continuous matrices. This dense structure formation is attributed to something. It is attributed to continuous pozzolanic reactions of fly ash in later stages. Its glass phase gradually dissociates. This occurs under long-term hydroxide ion attack. It releases active silicate ions. These react with residual calcium ions in the system. This generates secondary C-S-H. This reflects a delayed hydration process. It also reflects an extended activity period.

#### 2.2.2. Gel Filling-Induced Nanostructure Optimization and Density Changes

As shown in Figure 2e, the MIP-derived average pore diameter of the L3 group is lower than that of L0 throughout curing, decreasing to 52.5 nm at 28 days. Several MIP limitations must be acknowledged. First, MIP measures pore entrance diameters. It does not measure true pore body sizes. The “ink-bottle” effect causes overestimation in gel-rich systems [33,34]. Second, MIP cannot resolve gel porosity below 3.5 nm. This represents substantial porosity in C-S-H-rich systems. Third, reported porosities (L3: 31.9% at 28 d; L0: 22.6% at 28 d) exceed typical cement paste values. These should be interpreted as apparent MIP porosities only. They are useful for relative comparison between groups [33]. Subject to these limitations, the pore refinement effect originates from three synergistic aspects. First, PRBAs directly fill macropores. These PRBAs have particle sizes below 0.28 mm. The macropores are at the 100 to 1000 nm level. Second, directional growth of AFt crystals forms reinforcement layers. These form on pore walls. This compresses pore space. Third, continuous deposition of C-S-H gel gradually seals connected pores. This reduces L3 average pore diameter. It goes from 82 nm at 3 days to 52.5 nm at 28 days. The apparent porosity decreases from 35.6% at 3 days to 31.9% at 28 days. Compared to the L0 group, the L3 group shows greater decreases. The average pore diameter decreased by 115% more than L0, and porosity decreased by 29.4% more than L0. Notably, the L3 group bulk density significantly increases with curing time. Apparent density changes slightly. This indicates that solid waste replacement of natural aggregates does not introduce low-density defect phases. The inherent density of particles themselves basically remains unchanged. Macroscopic density improvement mainly originates from something. It originates from internal pore structure optimization. This occurs instead of phase composition changes. Meanwhile, smaller average pore diameter limits ion penetration. This improves chloride ion diffusion resistance. However, it also leads to flexural strength reduction. When stressed, crack propagation paths are extended. However, energy dissipation mechanisms are insufficient. This increases brittle fracture risk. Smaller average pore diameter and flexural performance deterioration macroscopic manifestations form micro-mechanisms, with 28-day strength increasing 12.1% compared to 14 days while E_a_ only slightly increases 2.7 kJ/mol. This is the phenomenon of later-stage hydration sacrificing partial mass transfer efficiency for higher structural integrity [33]. Despite these pore structure refinements, the MIP-derived porosity values remain apparent quantities; their decreasing trends across curing ages provide reliable evidence for matrix densification, but the absolute magnitudes should not be overinterpreted.

### 2.3. Hydration Reaction Process Thermal Behavior and Mechanism Analysis

#### 2.3.1. TG-DSC Analysis

As shown in Figure 3a, three groups of TG-DSC curves can be divided into four characteristic stages, but only the latter three temperature zones involve theoretical chemical changes. Therefore, subsequent decomposition activation energy calculations do not involve the first stage [26,34,35]. The first stage of 60 to 100 °C is surface adsorbed water desorption. Before TG testing, materials were dried. The temperature was 60 °C. The time was 48 h. This removed most residual adsorbed water on external surfaces. However, during TG testing, there is still considerable weight loss in this temperature zone. Therefore, adsorbed water desorption in this temperature zone can intuitively reflect the strong physical adsorption capability of C-S-H gel’s large specific surface area for water molecules, indirectly reflecting the complexity of material microstructure. Table 1 shows the results. Weight loss rates at 60 to 100 °C were observed. This was for different curing ages. The L0 group still shows high weight loss rates. This is consistent with previous findings. L0 hydration already tends to complete at 7 days of curing. The images in Figure 2c confirm this. They show large amounts of encapsulating C-S-H. Meanwhile, the L3 group weight loss in this temperature zone increases with curing time. This indicates its microstructure complexity. Ordinary low-temperature drying cannot completely remove adsorbed water. This is from its complex encapsulating structure. This also provides another piece of evidence: negative activation energy appears in the third stage.

(3) $$\begin{array}{c} nH_{2}O_{free/capillary}(l)\to nH_{2}O(g)↑ \end{array}$$

As shown in Figure 3a the second stage of 105–170 °C is C-S-H and AFt crystalline water removal. Crystalline water weight loss rates at 3 days are slightly higher than subsequent values, another manifestation of phosphogypsum’s water retention and retarding properties. Subsequent weight loss rates are all higher than the L0 group. This indicates that PRBAs generate more cementitious components than in traditional cement–sand systems. Meanwhile, weight loss rates increase with curing time. This reflects that hydration products can effectively improve strength and material density over long periods.

(4) $$\text{C-S-H}(s)\to CaO\cdot SiO_{2}(s)+H_{2}O(g)↑$$

(5) $$\begin{array}{c} {Ca}_{6}{\left[Al{\left(OH\right)}_{6}\right]}_{2}{\left(SO_{4}\right)}_{3}\cdot 26H_{2}O\left(s\right)\to {Ca}_{6}{\left[Al{\left(OH\right)}_{6}\right]}_{2}{\left(SO_{4}\right)}_{3}\left(s\right)+26H_{2}O\left(g\right)↑ \end{array}$$

The third stage of 405 to 450 °C theoretically involves something. It involves the preliminary removal of AFt structural hydroxyl. It also involves calcium hydroxide decomposition. These produce endothermic effects. However, TG curves present abnormal phenomena. No typical calcium hydroxide endothermic peak appears. DSC curves instead show weak exothermic peaks, with E_a_ calculation values of −7.2 to −10.1 kJ/mol, clearly indicating this is not a chemical decomposition process. SEM images in Figure 2d show that Ca(OH)_2_ crystals almost do not exist in the L3 system, being in a dynamic balance of “generation-consumption.” Meanwhile, this temperature zone weight loss process is exothermic. It is not endothermic. This is consistent with energy-level transition characteristics. These characteristics are of water molecule recombination after hydrogen bond breaking. Additionally, composite activator introduction brings large amounts of active sites. These are Si-OH and Al-OH sites. This gives C-S-H gel stronger physical adsorption capability. Therefore, weight loss at 405 to 450 °C is forced desorption of strongly physically adsorbed water. It is not calcium hydroxide decomposition. It should be noted that negative activation energy values alone cannot independently prove hydrogen bond breaking. Combined with DSC exothermic characteristics and calcium ion continuous consumption evidence chain, this process is confirmed. It is confirmed to be physical adsorption-dominated. However, it should be noted that physical adsorption dehydration exotherm is very obvious. It causes activation energy decrease. This possibly masks calcium hydroxide decomposition endothermic processes. Therefore, TG-DSC curves alone cannot indicate complete absence of calcium hydroxide in the L3 group. They only indicate that calcium hydroxide content is very low. It is less than the L0 group. Specific quantitative analysis requires additional characterization methods. The conclusion of “non-Ca(OH)_2_ decomposition” here only applies to explaining C-S-H structure complexification. The stepwise loss of AFt crystallization water in the 105–140 °C interval, yielding the partially dehydrated meta ettringite, is well-documented by Zhou and Glasser [36] and Pourchez et al. [37].

(6) $$\begin{array}{c} Ca{\left(OH\right)}_{2}(s)\to CaO(s)+H_{2}O(g)↑ \end{array}$$

The fourth stage of 650–730 °C is CaCO_3_ decomposition and complete AFt structure decomposition. The weight loss rate of 2.45% corresponds to CO_2_ uptake of 10.77 g/kg during ambient curing. This exceeds L0’s 1.96%. It reflects higher passive carbonation in the L3 system. This is attributable to L3’s greater porosity. It provides more surface area for atmospheric CO_2_ reaction. However, carbonation carries durability risks. It can reduce pH below 9. It may destabilize AFt and C-S-H. Shrinkage may introduce microcracking. Carbonation depth and pH monitoring are recommended for future work. Its E_a_ value drops sharply from 170.5 kJ/mol at 3 days to 125.8 kJ/mol at 14 days, indicating that carbonation generates high specific surface, low-crystallinity nanocrystals [38]. At temperatures exceeding 600 °C, the anhydrous calcium aluminate sulfate framework completely thermodynamically dissociates into its constituent oxides (CaO, Al_2_O_3_) and SO_3_ (g). Although direct experimental characterization of this specific dissociation for the anhydrous phases derived from AFt at high temperatures is still limited, this reaction is stoichiometrically consistent with the thermal decomposition behavior of the calcium aluminate sulfate system.

(7) $$\begin{array}{c} Ca{CO}_{3}(s)\to CaO(s)+CO_{2}(s)↑ \end{array}$$

(8) $$\begin{array}{c} {Ca}_{6}{Al}_{2}O_{6}{\left(SO_{4}\right)}_{3}\left(s\right)\to 6CaO\left(s\right)+{Al}_{2}O_{3}\left(s\right)+3SO_{3}(g)↑ \end{array}$$

Although nanostructures like C-S-H themselves may also undergo decarbonation reactions, calcium carbonate is the dominant phase in this temperature zone. C-S-H structures have already been destroyed and transformed before 600 °C. Meanwhile, C-S-H carbonation usually occurs on its surface. It also occurs between layers. Final products are still calcium carbonate. So, the two are not contradictory. Therefore, weight loss is mainly attributed to calcium carbonate crystal decomposition. Equation (10) shows the calculation. Calculating calcium carbonate size proves something. It proves nanocrystalline calcium carbonate generation during carbonation. The results are based on TG-DSC measured activation energy drop of 44.7 kJ/mol, combined with the surface energy theory formula.

(9) $$\begin{array}{c} ∆E_{a}=\frac{6\gamma V_{m}}{DN_{A}} \end{array}$$

Gamma is calcium carbonate surface energy. It is 1.2 J/m^2^. Vm is molar volume. It is 3.69 × 10^−5^ m^3^/mol. If crystallite size is XRD-measured 66 nm, theoretical activation energy drop is only 4.1 kJ/mol. However, TG-DSC-measured activation energy drop is 44.7 kJ/mol. This reversely calculates crystallite size to be approximately 6 nm.

This difference reveals significant size distribution heterogeneity. This is of carbonation products. XRD-measured 66 nm reflects something. It reflects the main coherent diffraction domain size. TG-DSC activation energy sharp drop reflects something else. It reflects contributions from small amounts of highly active nanocrystals. These ultra-fine crystallites may not be detected by XRD. This is due to low mass fraction or overly small crystallite size. However, they dominate thermal decomposition kinetic behavior. Together with 66 nm submicron matrix, they constitute a bimodal distribution carbonation product system. This “nano-submicron” composite structure ensures both matrix strength and passive atmospheric carbonation.

Age evolution patterns show that 3-day and 7-day TG curve morphologies are highly similar, with total weight loss rates of 6.8% and 7.2%, respectively, reflecting relatively single early hydration product types, mainly steel slag and cement hydration, and also indirectly reflecting the delayed hydration characteristics of the PRBA-based material. At 14 days, curves show four fluctuating weight loss segments. These are at 150 °C intervals. They are in the 150 to 600 °C temperature zone. This departs from traditional calcium hydroxide decomposition characteristics. Instead, it indicates C-S-H changing from single-layer to multi-layer complex structures. Inter-layer water molecules show graded desorption characteristics. This is due to binding energy differences. At 28 days, curves show intensified fluctuations. Weight loss slightly increases to 8.7%. This confirms later hydration tending to stabilize. However, structure continues to densify. Hydration products continuously increase.

#### 2.3.2. Thermal Behavior Differences Between Water-Washed and Selective Dissolution Samples

Water washing and selective dissolution are conventional means. They have been attempted in the literature for hydration product separation. They expose clear applicability limitations. These are in this ternary solid waste system. Figure 3b–d show the results. Although water washing treatment can remove residual calcium hydroxide, this is manifested as weight loss rate decrease. It decreases from 0.30% to 0.05% at 405 to 450 °C. TG curves show something else. Calcium hydroxide removal is accompanied by C-S-H gel damage. This shows strong age dependence. Since L3’s 405–450 °C weight loss temperature zone is caused by physical adsorbed water desorption, TG-DSC curves combined with calculations cannot distinguish between Ca(OH)_2_ decomposition and physical desorption.

Figure 3b shows the results. After water washing of 7-day aged specimens, an abnormal weight loss peak appears. It is 0.61% before 100 °C. This confirms that tubular C-S-H gel at this time still maintains complete capillary pore structure. It is capable of confining large amounts of free water through physical adsorption. This phenomenon indicates something. It indicates that early hydration products have structural tolerance. This is due to water washing treatment. TG curves still retain adsorbed water desorption characteristics.

Figure 3b shows the results. After water washing of 28-day age specimens, TG curves completely coincide with selective dissolution groups. The weight loss rate in the 405 to 450 °C interval is only 0.05%. No adsorbed water peak appears before 100 °C. This indicates that dense sheet-like C-S-H encapsulation layers undergo overall collapse. They also undergo detachment during water washing. This is due to mechanical disturbance and osmotic pressure mutation. This destruction causes C-S-H and AFt to coexist in agglomerate form. This makes subsequent selective dissolution unable to achieve phase separation.

This phenomenon reveals a methodological issue. The higher the densification degree, the more severe the failure. This is the failure of traditional chemical separation methods. This study therefore explicitly states something. For high hydration degree samples, water washing methods not only cannot effectively separate calcium hydroxide and C-S-H, but they also cause structural damage. Therefore, it is not recommended as a pretreatment method. This applies to complex cementitious systems.

### 2.4. Hydration Reaction Kinetic Modeling

#### 2.4.1. Reaction Model Fitting Goodness Comparison

Based on non-isothermal kinetic theory, fitting goodness tests were conducted on the L3 group characteristic weight loss intervals using Jander diffusion model, Avrami-Erofeev nucleation model (n = 2), and shrinking sphere model [24,39,40].

Table 2 shows the fitting results. The 115 to 140 °C interval is for C-S-H/AFt dehydration. The Jander model is optimal. Average R^2^ = 0.9980. This indicates the water molecule migration from nanopores and gel inter-layers are controlled by three-dimensional diffusion. The shrinking sphere model has average R^2^ = 0.9978 as the second best, revealing synergistic coupling between reaction interface advancement and diffusion mass transfer, consistent with a “diffusion-dominated, interface advancement-assisted” dual control mechanism. The control mechanisms of the various candidate models are compared in Table 3. This conclusion is consistent with SEM observations of multi-morphology C-S-H. It ranges from Type I tubular to Type II coral-like to sheet-like stacking coexistence. Different morphologies correspond to different dehydration energy barriers. This causes single models unable to completely describe the process. Both the Jander and shrinking sphere models yield R^2^ > 0.997 in the 115–140 °C interval. Careful justification is needed. The shrinking sphere model assumes uniform spherical interface contraction. This does not account for heterogeneous C-S-H pore structures. In contrast, Jander’s 3D diffusion model describes mass transport through porous media. It better represents water migration through C-S-H nanopore networks. Three observations support Jander selection. (1) Jander shows superior stability across 105–170 °C (R^2^ = 0.9226 vs. 0.9072). (2) SEM reveals multi-morphology C-S-H coexisting. This creates a heterogeneous diffusion field. (3) Water escaping through tortuous nanopores aligns with Jander’s framework. However, near-identical R^2^ values suggest a mixed mechanism. Future work should consider combined kinetic models [24,39,40]. The basic parameters of data fitting are summarized in Table 4. Jander’s three-dimensional diffusion equation better describes this cross-scale mass transfer process, while the sphere model only applies to geometric contraction of reaction interfaces, ignoring spatial heterogeneity of pore structure. Therefore, subsequent E_a_ calculations will adopt the Jander model as the final fitting result.

Table 2 shows the fitting results. The 405 to 450 °C interval is the ideal temperature zone. This is for calcium hydroxide decomposition. The Avrami model average R^2^ = 0.9973. The sphere model R^2^ = 0.9828. These are dual optimal fitting results. The Avrami-Erofeev model takes n = 2. This indicates that substances decomposing in this temperature zone grow in at least a two-dimensional manner. They exist as complex three-dimensional structures. However, activation energy calculation values are all negative. This clearly indicates that this process is not chemical decomposition. It is forced desorption of physically adsorbed water based on chemical changes.

Table 2 and Table 5 show the results. The 650 to 730 °C interval is for calcium carbonate decomposition. The Jander model is optimal. R^2^ = 0.9301. This is consistent with classical theory of carbon dioxide diffusion-controlled decomposition from carbonation product microcrystals. However, the L3 group weight loss rate of 2.45% in this interval is significantly higher than the L0 group’s 1.90%, and the E_a_ value drops sharply from 170.5 kJ/mol at 3 days to 125.8 kJ/mol at 14 days, revealing carbonation generates large amounts of nanoscale CaCO_3_ microcrystals. Microcrystals, due to large specific surface area and high surface energy, have reduced decomposition temperature and decreased activation energy, providing thermodynamic evidence for the material’s passive atmospheric carbonation. The linearized fitting curves are provided. They demonstrate optimal model correlations. These are for each temperature interval and they are in Supplementary Figure S2.

While R^2^ values provide a primary metric for goodness of fit, the near-identical correlation coefficients (>0.997) in the 115–140 °C interval necessitate additional statistical discrimination. Supplementary Table S6 presents the residual sum of squares (RSS), Akaike information criterion (AIC), Bayesian information criterion (BIC), and root-mean-square error (RMSE) for all candidate models. In the 115–140 °C interval, the Jander model maintains the highest R^2^ values across all curing ages (0.9968–0.9994, Table 2), and its three-dimensional diffusion framework is physically consistent with SEM-observed multi-morphology C-S-H nanopore networks. Although Avrami-Erofeev yields lower RSS in this interval, its AIC values are computed on a different linearized coordinate scale (g(α) = [−ln (1 − α)]^0.5^ versus Jander’s [1 − (1 − α)^1/3^]^2^), which inflates the total sum of squares and complicates direct cross-model AIC comparison. Consequently, model selection integrates (I) R^2^ stability, (II) physical mechanistic consistency with microstructural evidence, and (III) residual randomness validated in Supplementary Figure S4. For the 405–450 °C interval, Avrami-Erofeev is unambiguously optimal by both R^2^ (>0.99) and AIC/BIC minima. For the 650–730 °C interval, Jander is preferred because its R^2^ (0.8686–0.9724) substantially exceeds Avrami-Erofeev (0.7293–0.9077), indicating systematic bias in the latter. The Jander three-dimensional diffusion model was therefore adopted for activation energy calculations in all intervals where it is physically and statistically justified.

First, let α be the proportion of decomposition reaction completion during TG process. Then

(10) $$\begin{array}{c} \alpha =\frac{w_{0}-w_{T}}{w_{0}-w_{\infty }} \end{array}$$

$w_{0}$: Sample mass at decomposition starting temperature; unit: mg.
$w_{T}$: Sample mass at temperature T; unit: mg.
$w_{\infty }$: Sample mass after complete decomposition in temperature interval; unit: mg.

Let the mechanism function be f(α), with linear variation quantity g(α). In the Jander diffusion model equation:

(11) $$\begin{array}{c} g\left(a\right)={\left[1-{\left(1-\alpha \right)}^{\frac{1}{3}}\right]}^{2} \end{array}$$

(12) $$\begin{array}{c} Y=\ln\frac{g\left(a\right)}{T^{2}} \end{array}$$

In the Avrami-Erofeev (n = 2) diffusion model equation:

(13) $$\begin{array}{c} g\left(a\right)={\left(-\ln\left(1-\alpha \right)\right)}^{0.5} \end{array}$$

(14) $$\begin{array}{c} Y=\ln\frac{g\left(a\right)}{T^{2}} \end{array}$$

In the shrinking sphere model diffusion model equation:

(15) $$\begin{array}{c} g\left(a\right)=1-{\left(1-\alpha \right)}^{\frac{1}{3}} \end{array}$$

(16) $$\begin{array}{c} Y=\ln\frac{g\left(a\right)}{T^{2}} \end{array}$$

#### 2.4.2. Reaction Control Mechanism Analysis for Each Temperature Zone

**Figure 4:**
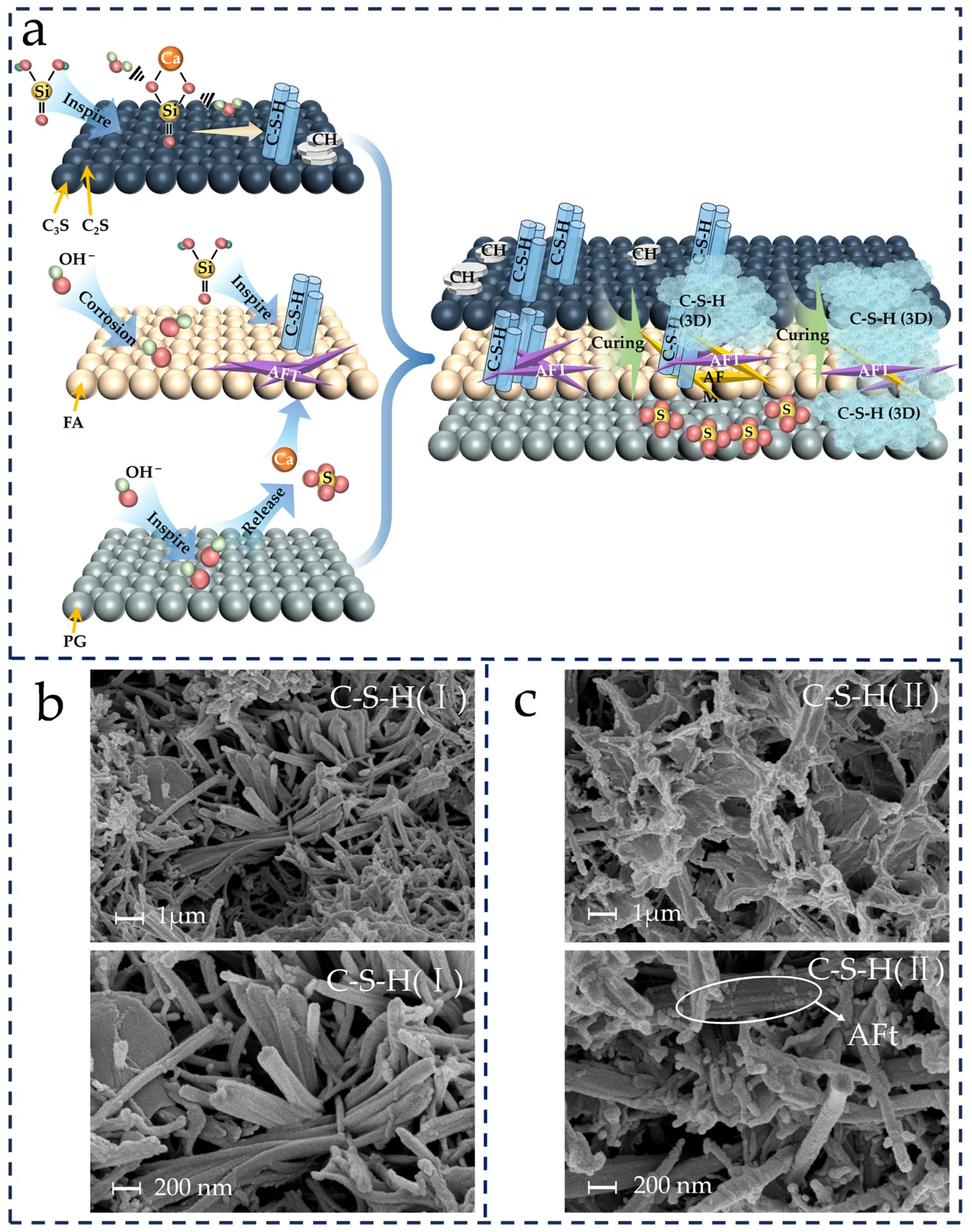
(**a**) Microscopic mechanism diagram of phosphogypsum, fly ash, and steel slag under alkali excitation; (**b**) SEM scan images of group L3 after 3 days of curing; (**c**) SEM scan images of group L3 after 28 days of curing.

Table 5, Figure 4, and Supplementary Figure S3 show the results. Temporal evolution of activation energy quantitatively reveals hydration product structure transformation pathways. Activation energy values in the 115 to 140 °C interval are as follows. At 3 days, it is 161.3 kJ/mol. It decreases to 130.1 kJ/mol at 7 days. It reaches the minimum value of 99.3 kJ/mol at 14 days. It slightly increases to 102.0 kJ/mol at 28 days. As shown in Figure 4, combined with SEM analysis, early E_a_ decrease is attributed to continuous outward growth of C-S-H structure, with water molecule diffusion paths optimized from “narrow-long” to “wide-short,” providing strong evidence for C-S-H changing from Type I tubular to Type II coral-like. Meanwhile, tube–tube bridging phenomena increase pore channels. This reduces dehydration resistance. Later activation energy recovery reveals dual effects. These are of fly ash later hydration. Continuously generated secondary C-S-H fills initial skeleton gaps. It reduces the average pore diameter to 62 nm. It also blocks some water molecules’ escape channels. This “structure densification–mass transfer complexification” trade-off relationship explains why 28-day strength increases 12.1% compared to 14 days while E_a_ only slightly increases 2.7 kJ/mol, indicating that mechanical performance improvement effects far exceed negative thermodynamic resistance effects. This phenomenon confirms the core assertion that “macroscopic structure stability and microscopic structure stability are negatively correlated,” i.e., later-stage hydration sacrifices partial mass transfer efficiency for higher structural integrity [27,28].

### 2.5. Phosphogypsum-Steel Slag-Fly Ash Synergistic Hydration Mechanism

The synergistic effect manifests as a temporal “relay race” hydration mechanism. This is conceptually illustrated in Figure 4a. This staged hydration behavior is further supported by recent comparative studies on phosphogypsum-fly ash-steel slag-stabilized soils, where the ternary composite binder modifies the load-bearing skeleton and fills pores through sequential physical and chemical reactions, outperforming Portland cement in both later-stage strength and durability under freeze-thaw and wet-dry cycles [41]. The schematic depicts staged contributions of each PRBA component. Steel slag provides early calcium initiation. Phosphogypsum supplies intermediate sulfate for AFt formation. Fly ash contributes later-stage densification via pozzolanic reactions. Figure 4a is a conceptual illustration. It should not be interpreted as a quantitative representation of spatial distributions or reaction rates [11,21,42].

Very Early Stage up to 3 days: Steel slag provides calcium initiation. C_2_S and C_3_S minerals in steel slag hydrate first. This occurs in the strong alkaline environment. It is provided by sodium silicate hydrolysis. Their hard porous skeleton not only provides physical support, it also regulates water distribution system. This occurs through capillary adsorption. It creates stable humidity environments for subsequent hydration. Calcium ions and hydroxide ions released at this stage rapidly establish high alkaline environments. This triggers early C-S-H nucleation. It also shortens fly ash depolymerization induction period. Simultaneous sodium silicate hydrolysis provides silicate ions. These rapidly react with calcium ions. They form nanoscale fibrous C-S-H networks. These provide anchoring sites for subsequent AFt crystal growth.

(17) $$\begin{array}{c} C_{2}S+2H_{2}O\to \text{C-S-H}+2Ca{\left(OH\right)}_{2} \end{array}$$

(18) $$\begin{array}{c} C_{3}S+3H_{2}O\to \text{C-S-H}+3Ca{\left(OH\right)}_{2} \end{array}$$

Early Stage 3 to 7 days: Phosphogypsum provides sulfur for AFt skeleton generation. As system calcium ion concentration reaches supersaturation, dihydrate gypsum in phosphogypsum begins slow sulfate release. This reacts with aluminum ions dissolved from steel slag and fly ash. It generates AFt crystals. AFt, as a key hydration gel product, enhances strength through gel binding and fixation; its hexagonal needle-like morphology interpenetrates into the gel network skeleton, forming unique “tube-sheet-bridge” multi-level interlocking structures. At this time, compressive strength first exceeds L0, validating the “strength skeleton” effect of sulfate activation.

(19) $$\begin{array}{c} {6Ca}^{2+}+2Al{\left(OH\right)}_{4}^{-}+3SO_{4}^{2-}+2OH^{-}+26H_{2}O\to {Ca}_{6}{\left[Al{\left(OH\right)}_{6}\right]}_{2}{\left(SO_{4}\right)}_{3}\cdot 26H_{2}O\left(s\right) \end{array}$$

Intermediate Stage 7 to 14 days: Fly ash depolymerization and secondary hydration occur. Cumulative hydroxide ions continuously attack fly ash glass phase surfaces. They break Si-O-Si and Al-O-Al bonds. They gradually release active silicon and aluminum components. Glass phase follows “surface erosion-interlayer stripping-bulk dissociation” kinetic processes under alkaline conditions. Released HSiO_3_^−^ migrates through three-dimensional diffusion to AFt skeleton gaps, reacting with residual Ca^2+^ to generate secondary C-S-H. At this stage, hydration products transform from tubular to sheet-like, with porosity decreasing from 35.2% to 33.9%, and gel layer thickness increasing, reflecting fly ash’s later-stage densification contribution.

(20) $$\begin{array}{c} {\left({Al}_{2}O_{3}\cdot 2SiO_{2}\right)}_{glass}+4OH^{-}+3H_{2}O\to 2HSiO_{3}^{-}+2Al{\left(OH\right)}_{4}^{-} \end{array}$$

Later Stage 14 days and beyond: Structure densification and continuous optimization occur. Micro-aggregate filling effects are significant. Figure 4b,c show the results. Residual fly ash continues hydration. C-S-H structure densifies. Tubular C-S-H roots connect into sheets. Morphology transforms to sheet-like and densely stacks. Secondary and tertiary hydration short tubular C-S-H begins growing. This occurs at distal ends of Type I C-S-H. This “low-E_a_ advantageous structure” formation reduces the average pore diameter to 52 nm, while the material shows passive atmospheric carbonation. Carbonation generates submicron fine-crystal CaCO_3_ with a main size of approximately 66 nm, and small amounts of highly active nanocrystals with sizes less than 10 nm, further densifying the matrix. Higher phosphogypsum ratios can continuously provide sufficient sulfate ions. This inhibits secondary hydration product AFm generation. This occurs through equilibrium reactions. It effectively controls delayed AFt expansion.

(21) $$\begin{array}{c} AFt⇌AFm+2SO_{4}^{2-} \end{array}$$

As depicted in Figure 4a, this relay mechanism proceeds through three stages. (1) Initial calcium supply (0–3 days): Steel slag provides Ca^2+^ through C_2_S/C_3_S hydration. (2) Intermediate skeletal formation (3–7 days): Phosphogypsum releases sulfate for AFt crystallization. (3) Ultimate densification (7–56 days): Fly ash mediates secondary C-S-H formation. Such staggered complementarity of activities enables something. It enables the L3 group to achieve a 12.1% increase in 28-day strength. This is compared to 14 days. Strength shows continuous growth to 12.47 MPa at 56 days. This thereby effectively overcomes the later-stage hydration attenuation bottleneck.

### 2.6. Environmental Stability and Harmful Element Solidification Mechanism

#### 2.6.1. Leaching Toxicity Results Analysis and Standard Comparison

As shown in Table 6, the L3 group 28-day P, F, and Mn leaching concentrations are 0.1203 mg/L, 0.0247 mg/L, and 0.4644 mg/L, respectively, satisfying the Class I discharge limits of GB 8978–1996 (P ≤ 0.5 mg/L, F ≤ 10 mg/L, Mn ≤ 2.0 mg/L). Moreover, the leaching concentrations are also below the thresholds specified for phosphogypsum-based road base materials in T/CPFIA 0010–2023 (phosphate as P ≤ 0.5 mg/L, fluoride as F ≤ 10 mg/L, determined by HJ 557) [42]. Compared to raw material phosphorus 0.362 mg/L and fluorine 0.211 mg/L leaching concentrations, solidification rates reach 66.7% and 88.3%, respectively. This result confirms something. It confirms that the multi-solid waste system converts soluble harmful ions into stable phases. This occurs through chemical precipitation and physical encapsulation dual mechanisms. Although Mn element leaching concentration reaches 0.4644 mg/L, steel slag raw material Mn background value is as high as 1.8046 mg/L, with the solidification rate also reaching 74.2%. Comparison with the reference thresholds for Class I general industrial solid waste as defined in GB 18599–2020 (standard for pollution control on the non-hazardous industrial solid waste storage and landfill) shows that the leaching concentrations of phosphorus, fluorine, and manganese in the L3 group are 24%, 0.25%, and 23% of the respective limits. The reference limits, derived from GB 8978–1996 (Class I discharge standard), are 0.5 mg/L for phosphorus, 10 mg/L for fluorine, and 2.0 mg/L for manganese. These results indicate that the environmental risks associated with the material are well controlled. The positive role of phosphogypsum in alkali-activated systems extends beyond sulfate activation; recent studies confirm that phosphogypsum addition can enhance heavy metal immobilization and reduce leaching risks in solidified matrices through combined chemical precipitation and physical encapsulation, corroborating the dual fixation mechanism identified herein [43].

#### 2.6.2. Ca^2+^-Dominated P/F Solidification Reaction Path and Microstructure Densification Barrier Effect on Pollutant Migration

Figure 2d and Figure 4a show the results. Harmful element solidification follows three-step paths. These are dissolution, precipitation, and encapsulation. In the dissolution stage, calcium phosphate and calcium fluoride in phosphogypsum slightly dissolve. This occurs in alkaline liquid phases. In the early hydration precipitation stage, sufficient Ca^2+^ reacts with PO_4_^3−^ to generate Ca_5_(PO_4_)_3_(OH) (K_sp_ = 2.3 × 10^−59^), and Ca^2+^ reacts with F^−^ to generate CaF_2_ (K_sp_ = 3.9 × 10^−11^) as difficult-to-dissolve precipitates [14].

(22) $$\begin{array}{c} 5{Ca}^{2+}+{3PO}_{4}^{3-}+4OH^{-}\to {Ca}_{5}{\left({PO}_{4}\right)}_{3}(OH)(s)↓ \end{array}$$

(23) $$\begin{array}{c} {Ca}^{2+}+{2F}^{-}\to CaF_{2}(s)↓ \end{array}$$

In the middle–late hydration encapsulation stage, this is closely combined with C-S-H morphology evolution. C-S-H densifies from 3-day tubular network structures to form 28-day sheet-like dense encapsulation layers. This morphology evolution gives gel phases stronger physical encapsulation capabilities, achieving “chemical fixation + physical encapsulation” synergistic stabilization. Meanwhile, pH of 12.4 of a high-alkaline environment ensures something. It ensures precipitation products remain undecomposed long-term. XRD detected calcium fluoride weak peaks in 28-day spectra. However, due to low diffraction intensity, they were not explicitly labeled. However, the 88% decrease in fluoride concentration in ICP constitutes indirect evidence. MIP-derived apparent porosity shows positive correlation trends with fluoride leaching concentration. These are between porosity and fluoride leaching concentration. The L3 group 28-day porosity of 31.9% decreases 3.3%. This is compared to 7 days. The 28-day average pore diameter of 52.5 nm decreases 20 nm. This is compared to 7 days. This corresponds to fluoride leaching decreasing. It goes from 0.041 to 0.0247 mg/L. This is a 39.8% decrease. In Figure S1, the potential applications of this material system include non-structural or semi-structural components, such as road bases, stabilized fill, and prefabricated non-load-bearing blocks. Applications requiring high strength, such as prefabricated box beams, could potentially be used after further improvement.

## 3. Conclusions

This study investigated a gel-based PRBA material system for road base applications. The ternary system uses phosphogypsum, fly ash, and steel slag. Four conclusions are drawn.

(1) Relay-race synergistic hydration. Steel slag provides early Ca^2+^. Phosphogypsum supplies intermediate sulfate for AFt. Fly ash densifies the matrix via pozzolanic reactions. L3 (55% PRBA, PG:FA:SS = 4:3:4) achieved 9.33 MPa at 28 days. This classifies it as road base material. The 22.0% increase over L0 reflects additional reactive binders. It does not reflect pure aggregate replacement.
(2) Non-isothermal kinetic characterization. C-S-H gel network/AFt dehydration follows 3D diffusion control (Jander, R^2^ = 0.998). E_a_ decreases from 161.3 to 99.3 kJ/mol at 14 days. The 405–450 °C interval shows negative E_a_. This is attributed to forced desorption of physically adsorbed water. Single heating rate TGA has limitations.
(3) Microstructural evolution. C-S-H gel network evolves from tubular gel (100–200 nm) to lamellar gel (<10 nm). Average pore diameter decreases from 82 to 52.5 nm. MIP porosity values (31.9% at 28 d) are apparent values affected by the ink–bottle effect and are suitable only for relative comparison between groups.
(4) Environmental performance. P and F solidification rates are 66.7% and 88.3%. Leaching concentrations satisfy GB 8978–1996 Class I limits. This gel-based material is designed for road base applications.
(5) Future directions. Long-term durability testing, extended carbonation monitoring, and fiber reinforcement for ITZ toughening are required to qualify this material for field road base deployment and broader civil engineering applications.

## 4. Materials and Methods

### 4.1. Raw Materials

The raw materials come from industrial enterprises in Yunnan Province, China. Phosphogypsum (PG) comes from a phosphate production plant. Steel slag (SS) comes from steel production facilities. Fly ash (FA) comes from coal-fired power plants. P.II 32.5 Portland cement is used as the base binder.

**Figure 5:**
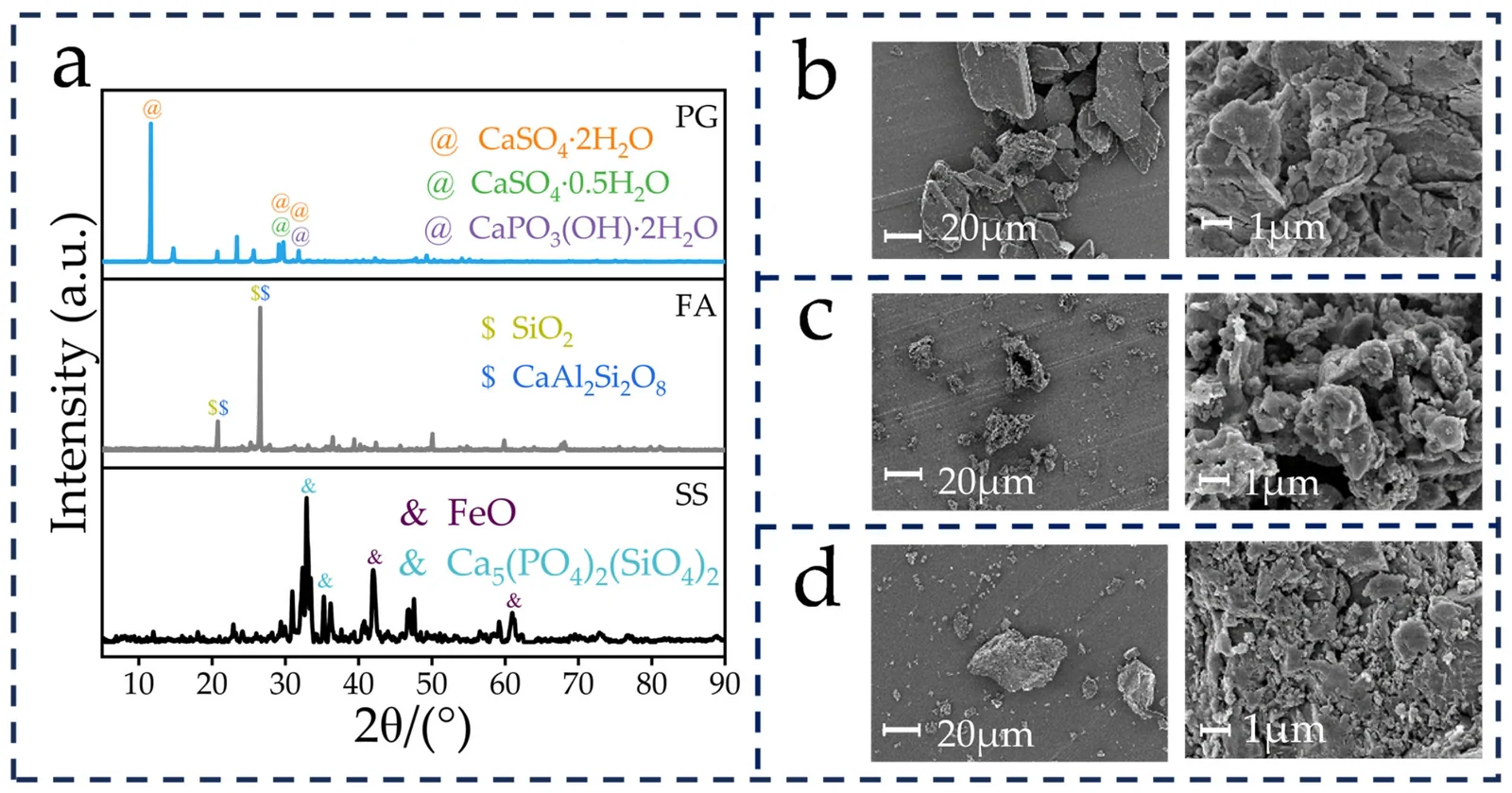
(**a**) XRD of the raw material; (**b**) SEM image of PG; (**c**) SEM image of FA; (**d**) SEM image of SS.

Phosphogypsum served as a high-sulfur active PRBA. It appeared as white powder. It contained small amounts of sand and gravel particles. As shown in Table 7 and Figure 5a, it contained 19.66% S and 27.27% Ca, possessing both fine aggregate filling effects and sulfate reserve functions. Its CaSO_4_·2H_2_O content can slowly release SO_4_^2–^ in alkaline environments, providing sulfur sources for subsequent AFt generation [44]. Although PG has relatively high leaching risks with P at 0.362 mg/L and F at 0.211 mg/L, its powder form facilitates conversion to stable phases through chemical–physical dual fixation.

Fly ash served as a high-silicon-aluminum active PRBA. Figure 5c and Table 7 show their characteristics. Fly ash appeared as gray spherical microbeads. It has significant ball-bearing effects. It also has micro-aggregate filling effects. Its silicon-aluminum-rich glass phase contained 24.98% Si and 11.27% Al, which can undergo pozzolanic reactions under composite activator action to generate secondary C-S-H gel for later-stage densification [44]. The spherical morphology of fly ash can improve workability. This applies to high solid waste systems. It compensates for flow loss. This loss is caused by angular phosphogypsum and steel slag particles. As a PRBA, fly ash optimizes particle gradation curves. It also achieves delayed hydration functionality. This occurs through long-term activity release.

Steel slag served as a high-calcium active PRBA. Figure 5d and Table 7 show its characteristics. Steel slag appeared as black powdery irregular particles after crushing. Its calcium content is as high as 29.42%. Rich in C_2_S, C_3_S, and f-CaO minerals, it possesses dual functions of early calcium supply and skeleton support [10,45]. The silicate skeleton of steel slag forms physical support in the later hardening stage; dissolved Ca^2+^ and OH^−^ provide alkaline environments and nucleation sites for early hydration. Steel slag contains 16.80% iron and 2.64% manganese. After alkaline environment stabilization, 28-day manganese ion leaching is only 0.4644 mg/L. The solidification rate is 74.2%. This meets environmental safety requirements for PRBAs.

Composite activator design. The above PRBAs have reaction temporal differences. To address these, a calcium hydroxide-sodium silicate composite gelation activator system was adopted. Calcium hydroxide was analytical grade. Its purity was 95% or higher. It provides hydroxide ions to attack fly ash glass phase. It also stabilizes hydration products. Sodium silicate pentahydrate was analytical grade. Its Na_2_O to SiO_2_ ratio was 1.03. It provides active silicate ions and sodium ions. These rapidly generate early C-S-H with calcium ions dissolved from steel slag. This shortens the induction period. Water serves as the primary solvent, while the composite activator (Ca(OH)_2_ + Na_2_SiO_3_·5H_2_O, 1:1 mass ratio, 2% of total PRBA mass) acts as the secondary solute providing reactive ions (OH^−^, SiO_3_^2−^) that initiate precursor dissolution and gelation through “alkali activation–sulfate activation” synergy [24,27,46,47]. It is emphasized that these alkali supplies are stoichiometrically consumed during C-S-H and AFt formation, thereby functioning as primary solutes in the activation chemistry rather than as catalysts that remain chemically unchanged.

Elemental analysis and leaching toxicity of raw materials are shown in Table 7. XRD and SEM characterization results are shown in Figure 5.

### 4.2. Experimental Design

#### 4.2.1. Mix Proportions

Preliminary experiments and literature review were conducted. Phosphogypsum content exceeding 25% results in non-setting. This occurs even after 24 h. Therefore, phosphogypsum was considered the core research focus. Fly ash and steel slag replacement rates were constrained. They did not exceed phosphogypsum content. Four replacement rates were selected for PRBA mix proportion optimization: 45% (L1, PG:FA:SS = 1:1:1), 50% (L2, 4:3:3), 55% (L3, 4:3:4; L4, 4:4:3), and 60% (L5, 1:1:1). The control group L0 used 100% natural river sand with 0% replacement. Table 8 presents the optimized mix proportions for formulations L0–L5. Water serves as the primary solvent, while the composite activator (Ca(OH)_2_:Na_2_SiO_3_·5H_2_O) functions as the secondary solute (2% of total PRBA mass) that provides OH^−^ and SiO_3_^2−^ ions to initiate precursor dissolution and gel formation. The binder-to-sand ratio was 0.3, and the water-to-binder ratio was 0.65 ± 0.05. All quantities are reported per 1 kg of total aggregate (natural sand + PRBA). All groups used a binder-to-sand ratio of 0.3 and a water-to-binder ratio of 0.65 ± 0.05. The composite activator (Ca(OH)_2_:Na_2_SiO_3_·5H_2_O = 1:1) was added at 2% of total PRBA mass. The boundary conditions for these formulations were identified from a complete 30-group preliminary experimental matrix, which is presented in Supplementary Table S1.

#### 4.2.2. Specimen Preparation and Aggregate Processing

Phosphogypsum was passed through a 50-mesh standard sieve. This removed lumpy phosphogypsum and other small gravel particles. Steel slag was crushed. It was then passed through a 50-mesh standard sieve. Specimen preparation followed the mixing, molding, and curing procedures described in GB/T17671–2021, which provides standardized methods for cement mortar strength testing. The binder-to-sand ratio was 0.3, and the water-to-binder (w/b) ratio was 0.65 ± 0.05. This reflects the actual experimental condition. The w/b was adjusted for each PRBA mix. This accounted for different water demands of phosphogypsum, fly ash, and steel slag. Consistent workability was ensured via slump measurement (10–20 mm). Slump is the correct workability indicator for concrete. It is important to clarify that this study investigates PRBA concrete for road base applications. Specimen dimensions of 160 mm × 40 mm × 40 mm follow GB/T17671–2021. This standardized prism method enables comparable strength evaluation for the ternary solid waste system. The composite activator consisted of analytical-grade calcium hydroxide (Ca(OH)_2_, purity ≥ 95%) and sodium silicate pentahydrate (Na_2_SiO_3_·5H_2_O, Na_2_O:SiO_2_ = 1.03). They were mixed at a 1:1 mass ratio. The activator was added at 2% of total PRBA mass. The complete experimental workflow, from raw material preparation to specimen fabrication and engineering application, is schematically illustrated in Figure S1.

Dry mixed materials were mixed in a cement mortar mixer. The mixing time was more than 90 s. Water was added in batches. The total duration was less than 90 s. The final total mixing time was controlled within 5 min. Specimen dimensions were 160 mm × 40 mm × 40 mm.

After reaching the specified curing time, specimens were dried in a 60–80 °C environment for 24 h to terminate hydration. Group L specimens underwent compressive and flexural strength testing, X-ray diffraction (XRD), X-ray fluorescence spectroscopy (XRF), scanning electron microscopy (SEM), inductively coupled plasma (ICP), thermogravimetric analysis (TG), and mercury intrusion prosimetra (MIP) [33,34,38].

### 4.3. Analytical Methods

#### 4.3.1. ICP-MS

An Agilent 7800 inductively coupled plasma mass spectrometer was used for sample analysis. Leachates of raw materials and specimens were prepared according to the “Solid Waste Extraction Procedure for Leaching Toxicity-Horizontal Vibration Method” (HJ 557–2010) to determine concentrations of P, F, Mn, Al, Pb, Cr, and other elements for environmental risk assessment. This method is widely used for comparative assessment of leaching behavior in solid waste-based materials and allows for direct comparison with regulatory thresholds. Hydration products were crushed to 200 mesh. They underwent selective dissolution treatment. This analyzed the feasibility of selective dissolution for C-S-H extraction. Leaching tests used a 1:100 liquid-solid ratio. They oscillated at 30 r/min for 8 h at 23 °C. Filtration followed through a 0.45 micrometer membrane. For selective dissolution, samples were placed in nitric acid solution. The pH was 3.0. The solid-liquid ratio was 1:100. The reaction time was 1 h. Water-washed control groups were set up simultaneously. This confirmed selective dissolution effectiveness.

#### 4.3.2. TGA

A synchronous thermal analyzer was used. The testing program heated uniformly from room temperature to 800 °C. The heating rate was 10 °C per minute. High-purity nitrogen protection was used throughout. The flow rate was 50 mL/min. Four theoretical characteristic temperature zones were focused on: 60–100 °C for adsorbed water desorption; 105–170 °C for C-S-H gel and AFt dehydration; 400–500 °C for Ca(OH)_2_ decomposition; 650–730 °C for CaCO_3_ decomposition [26,34,35].

### 4.4. Charactrization Methods

#### 4.4.1. XRD

An X-ray diffractometer was used with operating voltage of 40 kV, current of 40 mA, and scanning range of 2θ = 5–90° using Cu target. Sample preparation strictly followed cement-based material analysis specifications: specimens from each curing age were crushed and core regions selected, hydration was terminated with anhydrous ethanol, followed by vacuum drying at 60 °C for 48 h, crushing, grinding, and passing through a 200-mesh standard sieve [25,40,48]. All samples were crushed and ground to pass through a 200-mesh standard sieve (0.074 mm) to ensure representative and reproducible diffraction data.

#### 4.4.2. XRF

A Rigaku ZSX Primus III+ wavelength dispersive X-ray fluorescence spectrometer was used for chemical composition analysis of phosphogypsum, fly ash, and steel slag raw materials. Major elements Ca, S, Si, Al, Fe, and Mn and characteristic pollutant elements P and F were determined. Sample preparation used a powder pellet method with operating voltage of 50 kV, current of 60 mA, vacuum optical path environment, and scanning range of 2θ from 5 to 90°.

#### 4.4.3. SEM

A ZEISS Sigma 300 field emission scanning electron microscope was used. High-resolution images were collected at 5 kV accelerating voltage. For raw material characterization, trace amounts of powder were directly dispersed on conductive adhesive to observe original particle morphology. Specimens were dried at 60 °C for 24 h to terminate hydration, then crushed into approximately 1 cm^3^ pieces and gold-sputtered to enhance conductivity. Representative regions were selected during observation, and micro-regional morphology was systematically collected at magnifications of 5000× and 20,000× [38,49]. For cured specimens, hydration was terminated by drying at 60 °C for 24 h, followed by crushing into approximately 1 cm^3^ irregular fragments to expose fresh fracture surfaces for microstructural analysis. Hydration products were identified primarily by their characteristic morphologies following established SEM identification criteria for cementitious systems [49,50]: C-S-H gel appears as fibrous, tubular, or foil-like networks; calcium hydroxide (CH) presents as hexagonal platelets; and AFt exhibits needle-like or rod-like crystals. Steel slag (SS), due to its original form, is not powdery but rather in large granular form. Therefore, it was crushed before use, and the SEM observation is of the crushed steel slag.

#### 4.4.4. MIP

Pore structure characterization used a MICROMERITICS AutoPore IV 9500 mercury intrusion porosimeter. Specimen center portions were cut into approximately 5 mm × 5 mm × 10 mm pieces, and then vacuum dried at 60 °C for 72 h to constant weight to thoroughly remove evaporable water without damaging hydration product structure. Testing used continuous pressure mode, with low-pressure station range of 0.5–50 psi and high-pressure station range of 50–33,000 psi, totaling 130 pressure points with 10 s equilibrium time per point [1,33]. It should be noted that MIP-derived porosities are apparent values rather than absolute pore volumes. The technique measures pore entry diameters and is subject to the ink–bottle effect, particularly in gel-rich cementitious systems. Additionally, the method cannot resolve pore diameters below approximately 3.5 nm. Therefore, MIP data in this study are used exclusively for relative comparisons between formulations and curing ages.

## Figures and Tables

**Table 1:** Weight change in each core temperature zone.

Curing Time	3 d	7 d	14 d	28 d	3 d	7 d	14 d	28 d
temperature	60–110 °C				115–140 °C			
L0/(Δwt.%)	0.2531	1.1771	0.1984	0.1634	0.4153	1.5387	0.234	0.3436
L3/(Δwt.%)	0.4931	0.5777	1.0679	1.1500	1.956	1.725	1.846	2.0150
temperature	405–450 °C				650–730 °C			
L0/(Δwt.%)	0.1394	0.1273	0.2735	0.4152	1.5237	2.0127	1.7113	1.9016
L3/(Δwt.%)	0.0960	0.0826	0.1895	0.3014	2.0306	1.9868	2.1210	2.4502

**Table 2:** Goodness of fit of each model in each thermal weight loss region (R2).

Sample No.		L3–3	L3–7	L3–14	L3–28	Average Value
Temperature Range		105–170 °C				
Fitted Model	Jander	0.9042	0.9223	0.9281	0.9356	0.9226
	Avrami	0.8588	0.8782	0.8652	0.8835	0.8714
	Sphere	0.8895	0.9089	0.9102	0.9202	0.9072
Temperature Range		115–140 °C				
Fitted Model	Jander	0.9982	0.9994	0.9975	0.9968	0.9980
	Avrami	0.9982	0.9992	0.9957	0.9947	0.9970
	Sphere	0.9983	0.9993	0.9972	0.9978	0.9978
Temperature Range		405–450 °C				
Fitted Model	Jander	0.8952	0.9728	0.7526	0.7568	0.8444
	Avrami	0.9979	0.9994	0.9990	0.9929	0.9973
	Sphere	0.9900	0.9971	0.9958	0.9483	0.9828
Temperature Range		650–730 °C				
Fitted Model	Jander	0.9724	0.9627	0.9194	0.8686	0.9301
	Avrami	0.9077	0.8611	0.8221	0.7293	0.8301
	Sphere	0.9496	0.9235	0.8976	0.8375	0.9021

**Table 3:** Control mechanisms of various models.

Temperature Range	105–170 °C	115–140 °C	405–450 °C	650–730 °C
Optimal Model	Jander (R^2^ = 0.9226)	Jander (R^2^ = 0.9980)	Avrami–Erofeev (n = 2) (R^2^ = 0.9973)	Jander (R^2^ = 0.9301)
Ideal Model	Removal of AFt crystal water and dehydration decomposition of C-S-H		Ca(OH)_2_ decomposition and AFt hydroxyl removal	Decomposition of CaCO_3_ and complete decomposition of AFt
Actual reaction	Same as above		Physical desorption is dominant	Same as above
Core Mechanism	Diffusion–interface reaction combined control		Forced desorption of physically adsorbed water	CO_2_ Diffusion Control

**Table 4:** Basic parameters of data fitting.

Sample No.	Mass/(mg)			
	L3–3	L3–7	L3–14	L3–28
$w_{0}$	36.7780	51.4330	48.1960	50.4233
$w_{T=387.15K}$	36.5772	51.1025	47.2711	49.7903
$w_{T=443.15K}$	35.5878	49.7584	46.2817	48.2034
$w_{T=672.15K}$	35.3427	49.2947	45.8706	47.7442
$w_{T=721.15K}$	35.3074	49.2522	34.9109	48.5452
$w_{T=923.15K}$	34.9326	48.7944	45.3575	47.2660
$w_{T=1003.15K}$	34.1860	47.7690	44.3353	46.0330

**Table 5:** Table of activation energy values for each thermal weight loss region (E_a_).

Sample No.		L3–3	L3–7	L3–14	L3–28	Average Value
Temperature Range		105–170 °C				
Fitted Model	Jander	161.3072	130.1416	99.3381	101.9971	161.3072
	Avrami	36.9145	28.9025	21.2864	21.9728	36.9145
	Sphere	77.3248	61.7481	46.3411	47.6715	77.3248
Temperature Range		405–450 °C				
Fitted Model	Avrami	−9.8039	−10.0648	−8.6162	−7.2248	−9.8039
	Sphere	−8.4173	−8.9691	−6.4946	−4.1891	−8.4173
Temperature Range		650–730 °C				
Fitted Model	Jander	170.4879	150.8735	125.8204	133.3222	170.4879

**Table 6:** Leaching test of key elements in L3 group curing for 28 days.

Material	Concentration/(mg/L)				
	P	F	Mn	Al	Pb
L0	0.0028	0.0031	0.0023	3.8420	0.0098
L3	0.1203	0.0247	0.4644	0.1401	0.0055

**Table 7:** XRF analysis of certain key elements in the raw materials and ICP testing certain elements in raw material leachate.

Material	Mass Fraction/(%)			Material	Concentration/(mg/L)		
	PG	FA	SS		PG	FA	SS
P	0.42	0.12	0.91	P	0.362	0.073	0.573
F	0.40	0.00	0.00	F	0.211	0.002	0.001
Al	0.26	11.27	1.12	Mn	0.002	0.008	1.804
Ca	27.27	5.66	29.42	Al	9.469	0.095	0.048
S	19.66	0.64	0.12	Pb	0.004	0.005	0.004
Si	4.73	24.98	6.67	Cr	0.005	0.011	0.001

**Table 8:** Mix proportions of L0–L5 formulations (per 1 kg total aggregate).

Sample No.	Cement (g)	Natural Sand (g)	PRBA Replacement (%)	Mixing Ratio/(PG:FA:SS)	Water (mL)	Activator (g)
L0	300	1000	0	0	181	0
L1	300	550	45	1:1:1	187	9
L2	300	500	50	4:3:3	192	10
L3	300	450	55	4:3:4	196	11
L4	300	450	55	4:4:3	201	11
L5	300	400	60	1:1:1	208	12

## Data Availability

The raw data supporting the conclusions of this article will be made available by the authors on request.

## Supplementary Materials

The following supporting information can be downloaded at: https://www.mdpi.com/article/10.3390/gels12080726/s1, Figure S1: Engineering Application Approaches of Recycled Aggregate Concrete; Figure S2: Optimal fit result charts for each core temperature zone; Figure S3: Optimal fitted thermal decomposition energy of each core temperature zone; Table S1: Preliminary experiment mix proportions (30 groups); Table S2: Mix proportion of the specimen after determining the basic replacement range; Table S3: Brittleness index (B) of specimens at different ages; Table S4: Calculation of CaCO_3_ crystallite size based on Scherrer equation; Table S5: Calculation and verification of crystallite size based on surface energy theory; Table S6: Statistical criteria for kinetic model selection in each temperature interval.

## Abbreviations

The following abbreviations are used in this manuscript:

PRBA	powdered recycled binder-aggregate
PG	phosphogypsum
FA	fly ash
SS	steel slag
C-S-H	calcium-silicate-hydrate
AFt	AFt (calcium aluminate trisulfate hydrate)
SCM	supplementary cementitious material
C&D	construction and demolition
ITZ	interfacial transition zone
XRD	X-ray diffraction
XRF	X-ray fluorescence
SEM	scanning electron microscopy
ICP	inductively coupled plasma mass spectrometry
TG/TGA	thermogravimetric analysis
DSC	differential scanning calorimetry
MIP	mercury intrusion porosimeter
