# Supplementary material for: Gel-Dominated Microstructural Evolution and Strength Development in Phosphogypsum-Based PRBA Systems for Road Base Applications
Source: Gels. 2026 Aug 15;12(8):726. doi: 10.3390/gels12080726 (PMC13512482; doi:10.3390/gels12080726)
Supplement: Supplementary file 1 [file gels-12-00726-s001.zip › gels-4395700-supplementary.pdf]

Supplementary Information

Gel–Dominated Microstructural Evolution and Strength Development in Phosphogypsum–Based PRBA Systems for Road Base Applications

1. Introduction

This document provides additional experimental data and detailed characterization results. It supports the main manuscript. The materials included here are organized to assist readers. They help readers understand the comprehensive research methodology and results without overwhelming the main text.

2. Contents Overview

This supplementary file contains the following sections:

- Table S1: Preliminary experiment mixes proportions (30 groups)
- Table S2: Mix proportion of the specimen after determining the basic replacement range
- Table S3: Brittleness index (B) of specimens at different ages
- Table S4: Calculation of $CaCO_3$ crystallite size based on Scherrer equation
- Table S5: Calculation and verification of crystallite size based on surface energy theory

- Table S6: Statistical criteria for kinetic model selection in each temperature interval
- Figure S1: Engineering Application Approaches of Recycled Aggregate Concrete
- Figure S2: Optimal fit result charts for each core temperature zone
- Figure S3: Optimal fitted thermal decomposition energy of each core temperature zone

3. Table S1: Preliminary experiment mix proportions (30 groups)

3.1 Description

This table presents the preliminary experimental design. It comprises 30 mix proportion groups. The design systematically varies the replacement rates of phosphogypsum (PG), fly ash (FA), and steel slag (SS). This establishes the optimal ternary solid waste ratio range. The replacement rates for phosphogypsum were set at 15%, 20%, 25%, and 30%. Fly ash and steel slag contents were varied between 15% and 30%. This investigates their synergistic effects on workability. It also investigates effects on setting time. It investigates effects on early–age strength development. These preliminary trials provided critical boundary conditions. These conditions were for selecting the final experimental parameters. Parameters L1 to L5 are reported in the main manuscript. The binder–to–sand ratio was 0.3, and the water–to–binder ratio was 0.65±0.05 for all specimens.

3.2 Content to be Included

Table S1

Preliminary experiment mix proportions (30 groups)

Sample No.	Replacement rate/(wt.%)		
	PG	FA	SS
PG15–1	15	15	15
PG20–1	20	15	15
PG20–2	20	20	15
PG20–3	20	15	20
PG20–4	20	20	20
PG25–1	25	15	15
PG25–2	25	20	15
PG25–3	25	25	15
PG25–4	25	15	20
PG25–5	25	20	20
PG25–6	25	25	20
PG25–7	25	15	25
PG25–8	25	20	25
PG25–9	25	25	25
PG30–1	30	15	15
PG30–2	30	20	15

PG30–3	30	25	15
PG30–4	30	30	15
PG30–5	30	15	20
PG30–6	30	20	20
PG30–7	30	25	20
PG30–8	30	30	20
PG30–9	30	15	25
PG30–10	30	20	25
PG30–11	30	25	25
PG30–12	30	30	25
PG30–13	30	15	30
PG30–14	30	20	30
PG30–15	30	25	30
PG30–16	30	30	30

Note: Preliminary mixes with PG ≥30% exhibited non–setting within 24 hours and were excluded from further optimization. They are listed here only to document the full experimental matrix.

3.3 Purpose

The preliminary experiments were designed to achieve four goals. First, determine the upper limit of phosphogypsum content. Non–setting occurs beyond this limit. More than 25% causes failure within 24 hours. Second, identify the threshold

fly ash content. This prevents insufficient early strength. More than 30% causes significant strength reduction. Third, establish the critical steel slag content. This avoids excessive calcium ion generation. It also avoids low–activity calcium silicate precipitation. More than 30% consumes effective activator components. Fourth, narrow down the optimal replacement rate range. This range is 45% to 60%. It uses balanced phosphogypsum to fly ash to steel slag ratios. This is for subsequent systematic investigation. These boundary conditions directly informed the selection of L1 to L5 formulations. These are in Table 3 of the main manuscript.

4. Table S2: Mix proportion of the specimen after determining the basic replacement range

4.1 Description

This table presents the optimized mix proportions. These are for the formal experimental groups L0 to L5. They were established based on boundary conditions. These conditions were identified in the preliminary trials. These trials are in Table S1. Several determinations were made. Phosphogypsum content exceeding 25% induces non–setting phenomena. Fly ash content above 30% causes insufficient early strength. Steel slag content surpassing 30% leads to excessive calcium ion generation. It also leads to low–activity calcium silicate precipitation. Following these determinations, the final replacement rates were narrowed. The rates are 45%, 50%, 55%, and 60%.

The control group L0 utilizes natural river sand. It has 0% replacement. Experimental groups L1 to L5 employ ternary solid waste as recycled aggregates. These are phosphogypsum, fly ash, and steel slag. They use varying mass ratios. Specifically, L1 uses 45% replacement with ratio 1:1:1. L2 uses 50% replacement with ratio 4:3:3. L3 and L4 use 55% replacement with ratios 4:3:4 and 4:4:3 respectively. L5 uses 60% replacement with ratio 1:1:1. These formulations were designed to systematically investigate synergistic effects. These effects are of replacement rate and component ratio. They affect mechanical properties. They affect hydration kinetics. They affect microstructural evolution.

4.2 Content to be Included

Table S2

Mix proportion of the specimen after determining the basic replacement range.

Replacement rate/(wt.%)	Mixing ratio/ (PG: FA: SS)					
	L0	L1	L2	L3	L4	L5
0%	0:0:0					
45%		1:1:1				
50%			4:3:3			
55%				4:3:4	4:4:3	

60%	1:1:1

Note: L0 serves as the control group with natural river sand. L1–L5 represent recycled aggregate concrete with ternary solid waste (Phosphogypsum: Fly ash: Steel slag) at specified replacement rates and mass ratios. The binder–to–sand ratio was 0.3 for all mixes. The water–to–binder (w/b) ratio was adjusted within 0.65±0.05 to achieve a consistent slump of 10–20 mm, accounting for the different water demands of PG, FA, and SS.

4.3 Purpose

The inclusion of this mix proportion table serves to: (Ⅰ) document the systematic optimization process transitioning from the 30–group preliminary screening (Table S1) to the final 6–group formal experiment, providing reproducible experimental parameters; (Ⅱ) establish the comparative baseline between natural aggregate (L0) and multi–solid waste recycled aggregates (L1–L5), enabling quantitative assessment of the ternary system–s performance advantages; (Ⅲ) elucidate the design rationale for the optimal formulation L3 (55% replacement, PG:FA:SS = 4:3:4), which achieved the highest 28–day compressive strength (9.33 MPa) and extended hydration activity; and (Ⅳ) support the "relay race" mechanism analysis by correlating specific mix ratios (e.g., high SS in L3 for early Ca^{2+} supply, balanced PG for SO_4^{2-} release) with the temporal evolution of hydration products observed in SEM and XRD analyses. These data constitute the experimental foundation for the mechanical property trends

discussed in Section 3.1 and the kinetic modeling results presented in Section 3.4 of the main manuscript.

5. Table S3: Brittleness index (B) of specimens at different ages

5.1 Description

This table presents the calculated brittleness index. This is defined as the ratio of compressive strength to flexural strength. It is presented for control group L0. It is also presented for recycled aggregate groups L1 to L5. The curing ages are 3, 7, 14, 28, and 56 days. The brittleness index quantifies the material's tendency toward brittle fracture, with higher values indicating increased brittleness and reduced fracture toughness. The data reveal systematic differences between natural aggregate concrete L0 and recycled aggregate formulations. Particularly significant is the elevation of B values in L1 to L5 after the hydration peak, confirming the intrinsic brittleness introduced by powder recycled aggregates.

5.2 Content to be Included

Table S3

Brittleness index (B) of specimens at different ages

Sample No.	Curing Time/(d)				
	3	7	14	28	56
L0	1.67	1.98	1.83	2.01	2.38

L1	1.73	1.97	1.90	2.61	3.30
L2	1.83	2.43	2.06	2.60	3.30
L3	1.48	2.44	2.25	2.62	3.30
L4	0.88	1.56	1.82	2.60	3.30
L5	2.10	2.32	2.30	2.44	3.30

Note: B = $\frac{f_c}{f_{cf}}$, where f_c is compressive strength and f_{cf} is flexural strength.

5.3 Purpose

The brittleness index data serve to: (Ⅰ) quantitatively characterize the mechanical behavior divergence between high compressive strength and low flexural strength observed in recycled aggregate concrete; (Ⅱ) demonstrate that ITZ (interfacial transition zone) deficiencies introduced by powder recycled aggregates significantly increase material brittleness, with L3 reaching B = 2.62 at 28 days compared to L0's 2.01; (Ⅲ) provide evidence that optimized mix proportions (L3) cannot fully compensate for ITZ bonding deficiencies, as reflected by consistently higher B values than L0 throughout the curing period; and (Ⅳ) establish the need for fiber reinforcement or nano–modification strategies to improve fracture toughness in future engineering applications. These data support the main manuscript's discussion in Section 3.1.2 regarding flexural strength development and interface influence.

6. Table S4: Calculation of $CaCO_3$ crystallite size based on Scherrer equation

6.1 Description

This table details the application of the Scherrer equation. This equation calculates the crystallite size of calcium carbonate. The calcium carbonate is formed through carbonation. It is in the L3–28 specimen. The calculation utilizes X–ray diffraction data. This is from the (104) crystal plane reflection. Two theta is 29.447 degrees. Corrections are applied for instrumental broadening. Two methods of peak broadening analysis were employed. These are full width at half maximum and integral breadth. They yield crystallite sizes of approximately 143 nm and 66 nm respectively. The integral breadth method is less sensitive to peak shape anomalies. It provides the more reliable estimate. This estimate is 66 nm for the effective crystallite size.

6.2 Content to be Included

Table S4

Calculation of $CaCO_3$ crystallite size based on Scherrer equation

Crystal plane (hkl)	(104)
K	0.89
λ	0.15406nm

βinst	0.08°	
2θ	29.447°	
cosθ	0.967	
Broadening parameter	FWHM	Integral Breadth
Measured β	0.098°	0.146°
Corrected β	9.88×10^{-4}rad	2.13×10^{-3}rad
Crystallite size D	143nm	66nm

Note: The Scherrer equation: $D = K\lambda/(\beta\cdot\cos\theta)$, where β is the corrected peak broadening in radians.

6.3 Purpose

The Scherrer calculation serves to: (Ⅰ) quantify the nanocrystalline nature of carbonation products in the ternary solid waste system, confirming that $CaCO_3$ exists as sub–micron fine–crystals (66 nm) rather than conventional coarse crystals (>200 nm); Ⅱ) explain the pronounced peak broadening of the $CaCO_3$ (104) reflection (FWHM = 0.098°) observed in the XRD pattern, which reflects crystallite refinement rather than instrumental broadening; (Ⅲ) establish the primary crystallite size (66 nm) for subsequent surface energy theory calculations in Table S5; and (Ⅳ) provide structural evidence for the material's enhanced carbon sequestration capability (10.77 g/kg) and

improved matrix densification through nano–filling effects. These data directly support the discussion in Section 2.2.1 of the main manuscript regarding carbonation product characteristics.

7. Table S5: Calculation and verification of crystallite size based on surface energy theory

7.1 Description

This table presents an independent verification. It verifies calcium carbonate crystallite size. It uses surface energy theory. This is based on the activation energy drop measured by thermogravimetric–differential scanning calorimetry. The theoretical framework relates the reduction in decomposition activation energy. This relates to the increased surface energy of nanocrystalline materials. The measured activation energy drop is 44.7 kJ/mol. This is from the 650 to 730°C decomposition interval. The calculated crystallite size is approximately 6 nm. This contrasts with the 66 nm value from XRD Scherrer analysis. This discrepancy reveals a bimodal size distribution. Sub–micron crystals of 66 nm constitute the matrix. Ultra–fine nanocrystals of 6 nm dominate thermal kinetic behavior.

7.2 Content to be Included

Table S5

Calculation and verification of crystallite size based on surface energy theory

γ	V_m	ΔE_a	D
1.2 J/m^2	3.69×10^{-5}m^3/mol	4.1 kJ/mol	65.0nm
		44.7 kJ/mol	5.9nm

Surface energy theory equation: $\Delta E_a = 6\gamma V_m/(DN_A)$

For comparison: XRD Scherrer result D ≈ 66 nm yields theoretical ΔE_a = 4.1 kJ/mol

ΔE_a = 44.7 kJ/mol corresponds to the decrease in E_a for the 650–730 °C decomposition interval between 3–day and 14–day cured L3 specimens (see Table 6 in main text).

7.3 Purpose

The purpose of surface energy calculation is to: (Ⅰ) verify the presence of ultrafine nanocrystalline $CaCO_3$ (<10 nm), which, despite their low mass fraction, have a significant impact on the kinetics of thermal decomposition; (Ⅱ) reconcile the apparent contradiction between the grain size measured by XRD (66 nm) and the decrease in activation energy measured by TG–DSC (44.7 kJ/mol), indicating that the carbonation products exhibit a bimodal "nano–submicron" composite structure; (Ⅲ) confirm that nanocrystals of approximately 6 nm, although difficult to accurately detect via XRD due to low concentration or broad peaks, play a dominant role in the CO_2 diffusion–controlled decomposition process; and (Ⅳ) provide thermodynamic evidence for the excellent carbon capture capability of the phosphogypsum–based system, where high specific surface area nanocrystals both ensure the matrix strength and maintain active carbonation capacity. These findings support the "low E_a advantageous structure" mechanism discussed in Section 2.3.1 of the main text.

8. Table S6: Statistical criteria for kinetic model selection in each temp erature interval

8.1 Description

This table presents comprehensive statistical criteria for evaluating and selecting the optimal kinetic model across three characteristic thermal decomposition intervals: 115–140 °C (C–S–H/AFt dehydration), 405–450 °C (AFt hydroxyl removal/physical desorption), and 650–730 °C ($CaCO_3$/AFt complete decomposition). For each temperature interval and curing age (L3–3, L3–7, L3–14, L3–28), three candidate models–Jander three–dimensional diffusion, Avrami–Erofeev (n=2), and shrinking sphere–are compared using four statistical metrics: residual sum of squares (RSS), root–mean–square error (RMSE), Akaike information criterion (AIC), and Bayesian information criterion (BIC). The coefficient of determination R^2 values is reproduced from Table 3 of the main manuscript for direct comparison. The sample size n for each linearized fit is also reported. These metrics collectively address the limitation of relying solely on R^2 when correlation coefficients exceed 0.997, providing rigorous statistical discrimination for model selection..

8.2 Content to be Included

Table S6

Statistical criteria for kinetic model selection in each temperature interval.

Temperature Range	Sample	Fitted Model	n	RSS	RMSE	AIC	BIC
115–140°C	L3–3	Jander	167	0.20233	0.03481	–1117.55	–1111.31
		Avrami	167	0.01198	0.00847	–1589.60	–1583.36
		Sphere	167	0.05087	0.01745	–1348.11	–1341.88
	L3–7	Jander	167	0.05228	0.01769	–1343.55	–1337.31
		Avrami	167	0.00325	0.00441	–1807.46	–1801.23
		Sphere	167	0.01315	0.00887	–1574.04	–1567.80
	L3–14	Jander	167	0.12003	0.02681	–1204.75	–1198.51
		Avrami	167	0.00956	0.00757	–1627.28	–1621.05
		Sphere	167	0.02953	0.0133	–1438.94	–1432.70
	L3–28	Jander	167	0.15948	0.0309	–1157.29	–1151.05
		Avrami	167	0.01233	0.00859	–1584.79	–1578.55
		Sphere	167	0.03938	0.01536	–1390.87	–1384.63
405–450°C	L3–3	Jander	273	0.00864	0.00563	–2824.51	–2817.29
		Avrami	273	0.00054	0.00141	–3581.42	–3574.20
		Sphere	273	0.00196	0.00268	–3229.49	–3222.27
	L3–7	Jander	271	0.00302	0.00334	–3086.65	–3079.45
		Avrami	266	0.00016	0.00077	–3812.87	–3805.71
		Sphere	271	0.00064	0.00154	–3507.12	–3499.92
	L3–14	Jander	271	0.00163	0.00245	–3253.77	–3246.57

		Avrami	271	0.00021	0.00088	–3809.11	–3801.91
		Sphere	271	0.00049	0.00134	–3579.49	–3572.29
		Jander	272	0.00944	0.00589	–2789.06	–2781.85
	L3–28	Avrami	272	0.00101	0.00193	–3396.98	–3389.77
		Sphere	271	0.00258	0.00309	–3129.32	–3122.12
		Jander	483	3.56269	0.08588	–2367.29	–2358.93
	L3–3	Avrami	483	0.63714	0.03632	–3198.67	–3190.31
		Sphere	483	0.8608	0.04222	–3053.35	–3044.99
		Jander	485	3.94121	0.09015	–2330.14	–2321.77
	L3–7	Avrami	485	0.64738	0.03653	–3206.20	–3197.83
		Sphere	485	0.95201	0.0443	–3019.16	–3010.80
650–730℃		Jander	482	5.97411	0.11133	–2112.23	–2103.87
	L3–14	Avrami	482	1.14173	0.04867	–2909.88	–2901.53
		Sphere	482	1.4799	0.05541	–2784.84	–2776.48
		Jander	484	11.6338	0.15504	–1800.43	–1792.07
	L3–28	Avrami	484	2.34901	0.06967	–2574.80	–2566.43
		Sphere	484	2.88992	0.07727	–2474.49	–2466.13

Note: AIC/BIC values are computed based on the linearized coordinate $Y = \ln[g(\alpha)/T^2]$. Due to differing numerical ranges of the mechanism function $g(\alpha)$ across models, direct cross–model AIC comparison should be interpreted alongside adjusted R^2 and physical mechanistic consistency.

8.3 Purpose

This table serves to: (Ⅰ) provide rigorous statistical evidence beyond R^2 for kinetic model selection, as requested by the reviewers, when correlation coefficients exceed 0.997 and conventional goodness–of–fit metrics become insufficiently discriminatory; (Ⅱ) demonstrate that in the 115–140 °C interval, the Jander model maintains the highest R^2 values across all curing ages (0.9968–0.9994, Table 3), supporting its selection for C–S–H/AFt dehydration despite near–identical correlation coefficients; (Ⅲ) unambiguously confirm the superiority of the Avrami–Erofeev model in the 405–450 °C interval through both R^2 (>0.99) and AIC/BIC minima; and (Ⅳ) statistically justify the selection of the Jander model in the 650–730 °C interval, where its R^2 (0.8686–0.9724) substantially exceeds that of Avrami–Erofeev (0.7293–0.9077), revealing systematic bias in the latter. These data substantiate the kinetic modeling conclusions presented in Section 2.4 of the main manuscript.

9. Figure S1: Engineering Application Approaches of Recycled Aggregate Concrete

9.1 Description

This figure presents a schematic illustration demonstrating the practical engineering implementation pathway of the developed phosphogypsum–based material.

The diagram establishes a clear technological trajectory from laboratory–scale material preparation to civil infrastructure applications. It systematically depicts five critical stages: (Ⅰ) raw material preparation utilizing industrial solid wastes (phosphogypsum, fly ash, and steel slag) as PRBAs; (Ⅱ) composite activation employing sodium silicate–calcium hydroxide binary activators; (Ⅲ) standardized specimen preparation through mixing, molding, and controlled curing; (Ⅳ) deployment in primary engineering scenarios suitable for road base materials; and (Ⅴ) potential applications in prefabricated non–structural components after further improvement.

It is important to clarify that the material's 28–day compressive strength (9.33 MPa) classifies it as a road base or stabilized fill material. Applications requiring high strength, such as prefabricated box beams, could potentially be used only after further development and validation.

9.2 Content to be Included

Figure S1

Engineering Application Approaches of Recycled Aggregate Concrete

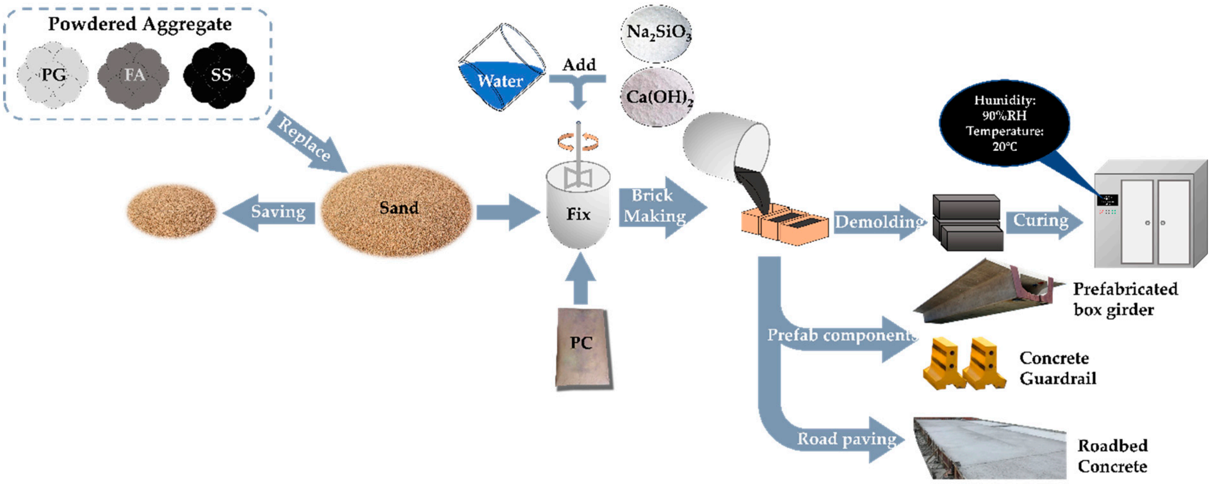

Note: The current material is not recommended for high–strength applications, such as precast box girders. However, it is expected that further strengthening and durability testing could make it feasible.

9.3 Purpose

This schematic serves to: (Ⅰ) visually demonstrate the complete technological chain from waste raw materials to high–value engineering products, validating the practical applicability of the laboratory–developed material system; (Ⅱ) illustrate specific civil engineering deployment scenarios where the material meets relevant technical standards, particularly emphasizing its suitability for prefabricated components and road infrastructure; (Ⅲ) provide tangible evidence supporting the environmental benefit claims by showing large–scale consumption pathways for phosphogypsum and other industrial solid wastes; (Ⅳ) establish a reference framework for subsequent engineering trials and commercialization pathways; and (Ⅴ) complement the mechanistic and kinetic analyses presented in the main

manuscript by contextualizing the scientific findings within real–world application scenarios.

10. Figure S2: Optimal fit result charts for each core temperature zone

10.1 Description

This figure presents the optimal kinetic model fitting results. These are for thermal decomposition of L3 specimens. They cover three characteristic temperature intervals. The first is 105 to 170 °C for C–S–H/AFt dehydration. The second is 405 to 450 °C for calcium hydroxide decomposition and physical desorption. The third is 650 to 730 °C for calcium carbonate decomposition. The Jander three–dimensional diffusion model demonstrates superior fitting goodness. R^2 is greater than or equal to 0.998. This is for the 115 to 140 °C interval. The Avrami–Erofeev model with n=2 optimally describes the 405 to 450 °C interval. The 650 to 730 °C interval follows Jander diffusion control. This is consistent with carbon dioxide diffusion–limited decomposition. These fittings establish the mechanistic basis. This basis is for activation energy calculations.

10.2 Content to be Included

Figure S2

Optimal fit result charts for each core temperature zone

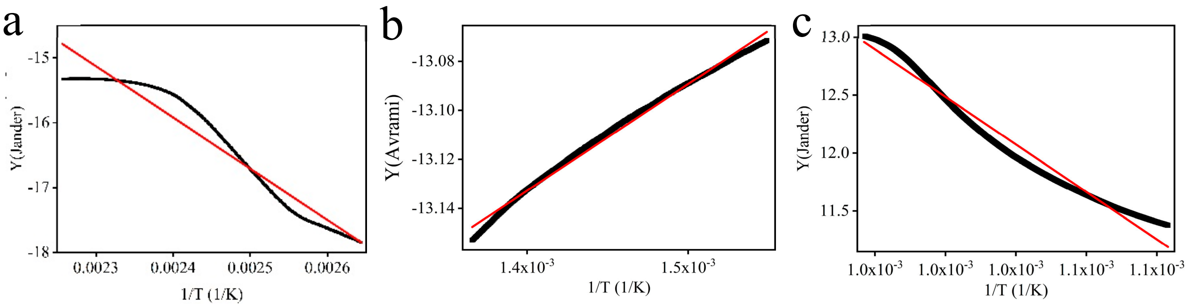

(a) Schematic diagram of the fitting situation in the 105–170°C weight loss temperature zone.

(b) Schematic diagram of the fitting situation in the 405–450°C weight loss temperature zone.

(c) Schematic diagram of the fitting situation in the 650–730°C weight loss temperature zone.

10.3 Purpose

These fitting charts serve four purposes. First, they visually demonstrate the superior correlation coefficients of the Jander model. These are for C–S–H/AFt dehydration with R^2 of 0.9980. They are also for calcium carbonate decomposition with R^2 of 0.9301. This validates the three–dimensional diffusion control mechanism. Second, they illustrate the exceptional fit of the Avrami–Erofeev model. This is with n=2 and R^2 = 0.9973. It is for the 405 to 450 °C interval. This indicates two–dimensional growth of complex three–dimensional structures. Third, they provide graphical evidence. The 115 to 140 °C narrow interval exhibits more stable fitting. This is

compared to the broader 105 to 170 °C range. This justifies the selection of specific temperature windows. These are for precise kinetic analysis. Fourth, they support the mechanistic interpretations. These are in Table 7 and Figure 5 of the main manuscript. They regard reaction control mechanisms and their temporal evolution.

11 Figure S3: Optimal fitted thermal decomposition energy of each core temperature zone

11.1 Description

This figure illustrates the temperature–dependent activation energy profiles. These are for L3 specimens. They cover different curing ages. The ages are 3, 7, 14, and 28 days. These profiles are derived from the optimal kinetic model fittings. The visualization highlights the temporal evolution of activation energy. This is in the 115 to 140 °C interval. It shows the characteristic decrease from 161.3 kJ/mol at 3 days to 99.3 kJ/mol at 14 days. This is followed by slight recovery to 102.0 kJ/mol at 28 days. The figure also depicts the anomalous negative activation energy values. These range from -7.2 to -10.1 kJ/mol. They are in the 405 to 450 °C interval. This confirms physical desorption rather than chemical decomposition. It also shows the sharp activation energy drop in the 650 to 730 °C interval. This drop is from 170.5 to 125.8 kJ/mol. It indicates nanocrystalline calcium carbonate formation.

11.2 Content to be Included

Figure S3

Optimal fitted thermal decomposition energy of each core temperature zone

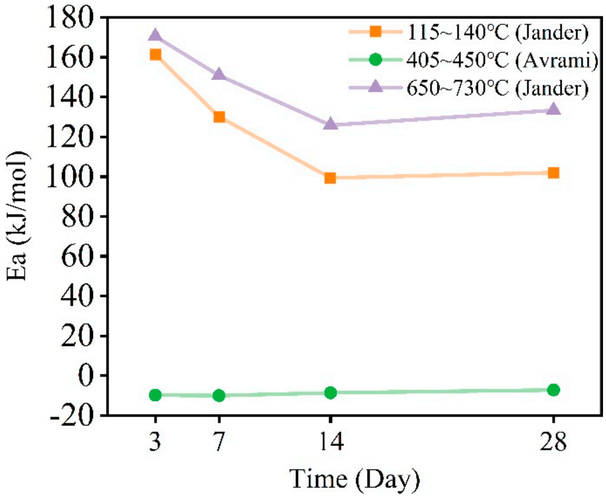

11.3 Purpose

This figure serves to: (Ⅰ) graphically demonstrate the "low–E_a advantageous structure" formation mechanism, where the minimum E_a at 14 days coincides with optimal C–S–H morphology transition from tubular to sheet–like structures; (Ⅱ) validate the negative correlation between macroscopic structural stability (strength increase) and microscopic mass transfer efficiency (E_a decrease), as 28–day strength improves 12.1% while E_a only increases 2.7 kJ/mol from the minimum; (Ⅲ) provide visual evidence for the bimodal carbonation product distribution through the discrepancy between XRD–derived and TG–DSC–derived crystallite sizes; and (Ⅳ) support the relay–race synergistic mechanism by showing how E_a evolution reflects the staged contribution of steel slag (early E_a decrease), phosphogypsum (intermediate stabilization), and fly ash (late–stage densification). These data underpin the kinetic modeling conclusions. These are in Section 3.4 of the main manuscript.

12. Summary

This Supplementary Information provides comprehensive supporting data for the main manuscript. Figure S1 establishes the engineering application pathway, demonstrating the practical implementation of phosphogypsum–based gel material system from laboratory preparation to large–scale civil infrastructure including prefabricated box girders, concrete guardrails, and roadbed concrete. Table S1 establishes boundary conditions for mix design through preliminary screening; Table S2 documents the optimized L0–L5 formulations; Table S3 quantifies the elevated brittleness index due to ITZ deficiencies; Tables S4–S5 resolve the bimodal "nano–sub–micron" carbonation structure via XRD and surface energy theory; while Figures S2–S3 validate the non–isothermal kinetic models. Collectively, these substantiate the "relay race" synergistic mechanism and "low–E_a advantageous structure" central to phosphogypsum–based multi–solid waste utilization, while confirming the gel material's engineering viability for sustainable infrastructure development.
